# From Gut Microbiota to Hepatic Pre-Metastatic Niches: Mechanism and Translational Prospects of the Gut–Liver Axis in Regulating Colorectal Cancer Liver Metastasis

**DOI:** 10.3390/microorganisms14092032

**Published:** 2026-09-12

**Authors:** Shiyi Wang, Jiachao Wang

**Affiliations:** 1 School of Basic Medicine, Hebei Medical University, Shijiazhuang 050017, China; 2 Key Laboratory of Immune Mechanism and Intervention on Serious Disease in Hebei Province, Department of Immunology, Hebei Medical University, Shijiazhuang 050017, China

**Keywords:** colorectal-cancer, liver metastasis, gut–liver axis, intestinal microbiome, immune remodeling

## Abstract

Colorectal cancer (CRC) is one of the most common malignancies worldwide, and liver metastasis remains a major contributor to poor prognosis and treatment failure. Increasing evidence indicates that the gut–liver axis plays a critical role in colorectal cancer liver metastasis (CRLM) by linking intestinal microbial alterations with hepatic microenvironmental remodeling. Gut dysbiosis and intestinal barrier disruption facilitate the translocation of microbial components and metabolites through the portal circulation, contributing to hepatic immune remodeling, extracellular matrix alteration, and the establishment of a pre-metastatic niche favorable for tumor colonization. During metastatic progression, specific tumor-associated microorganisms may further promote circulating tumor cell (CTC) survival, immune evasion, and metastatic adaptation, while intratumoral microbiome alterations and metabolic reprogramming may influence tumor growth and therapeutic responses. This review summarizes the current understanding of gut–liver axis-mediated regulation of CRLM, focusing on intestinal barrier dysfunction, microbial translocation, hepatic pre-metastatic niche formation, tumor cell–microbiota interactions, and emerging clinical applications. Unlike previous reviews that have primarily focused on gut microbiota alterations in colorectal carcinogenesis, this review emphasizes the contribution of gut-derived microbial signals to liver-specific metastatic evolution. In addition, we discuss current challenges, including limited human causal evidence, technical issues in low-biomass microbiome analysis, and the need for longitudinal clinical validation. Future integration of spatial multiomics, prospective cohorts, and personalized microbiome-based interventions may provide new opportunities for early risk prediction and precision management of CRLM.

## 1. Introduction

Colorectal cancer (CRC) ranks among the malignant tumors with the highest global incidence and mortality rates, with the liver being the most prevalent and clinically significant site of distant metastasis in CRC. The occurrence of liver metastasis not only significantly reduces long-term survival but also represents a major cause of treatment failure and disease-related mortality. Large population-based studies have demonstrated that a considerable proportion of CRC patients present with synchronous liver metastasis at diagnosis, while a subset of patients undergoing radical resection subsequently develop metachronous liver metastasis during follow-up [1]. Based on the timing of occurrence, colorectal cancer liver metastasis (CRLM) can be classified into synchronous liver metastasis, early metachronous liver metastasis, and late metachronous liver metastasis, with synchronous disease generally associated with higher tumor burden and poorer clinical outcomes [2]. Although hepatectomy, local ablation, systemic chemotherapy, targeted therapy, and emerging treatment strategies have improved survival for selected patients, durable disease control remains challenging [3]. One important reason is that current therapeutic strategies mainly target established metastatic lesions, whereas the mechanisms underlying hepatic microenvironmental preparation before tumor cell arrival, circulating tumor cell (CTC) colonization, and metastatic outgrowth remain incompletely understood [4,5,6].

The “seed and soil” hypothesis provides a classical theoretical framework for understanding organ-specific metastasis. According to this concept, successful metastatic colonization depends not only on the invasive and migratory properties of tumor cells (“seeds”) but also on whether the target organ provides a supportive microenvironment (“soil”) that allows tumor cell survival, adhesion, and proliferation. Traditional studies of CRLM have mainly focused on tumor cell-intrinsic mechanisms, including epithelial–mesenchymal transition, vascular invasion, extracellular matrix remodeling, and angiogenesis. However, increasing evidence indicates that CRLM is not solely determined by tumor cell characteristics but represents a complex ecological process involving interactions among tumor cells, immune cells, stromal components, metabolic signals, and microbial factors [7,8,9,10,11].

The gut–liver axis represents a critical anatomical and functional connection between intestinal microorganisms and hepatic homeostasis. Unlike liver metastases originating from many extra-intestinal malignancies, CRC has a unique anatomical relationship with the liver through portal circulation, allowing gut-derived microbial components, metabolites, and inflammatory signals to directly influence the hepatic microenvironment [12]. Under physiological conditions, the liver maintains a balance between immune surveillance and immune tolerance through coordinated regulation by Kupffer cells, hepatic sinusoidal endothelial cells, hepatic stellate cells, and other immune populations. During CRC progression, gut microbiota dysbiosis and disruption of intestinal barrier integrity may promote the abnormal translocation of bacteria and microbial-derived molecules into the portal circulation. Continuous exposure of the liver to these gut-derived signals can induce inflammatory responses, immune remodeling, and extracellular matrix alterations, thereby contributing to the establishment of a pre-metastatic microenvironment favorable for tumor colonization.

Beyond the formation of the pre-metastatic niche, gut microbiota may also participate in subsequent stages of CRLM progression. Specific tumor-associated microorganisms, such as *Fusobacterium nucleatum*, have been reported to influence tumor cell behavior through bacteria–tumor interactions, promoting tumor progression, immune evasion, and metastatic adaptation. In addition, alterations in microbial composition and metabolism within primary tumors and metastatic lesions may further affect metastatic growth, immune tolerance, and therapeutic responses. These findings suggest that gut microbiota may function not merely as a biomarker of disease progression but also as an active regulator of the metastatic ecosystem.

**Figure 1:**
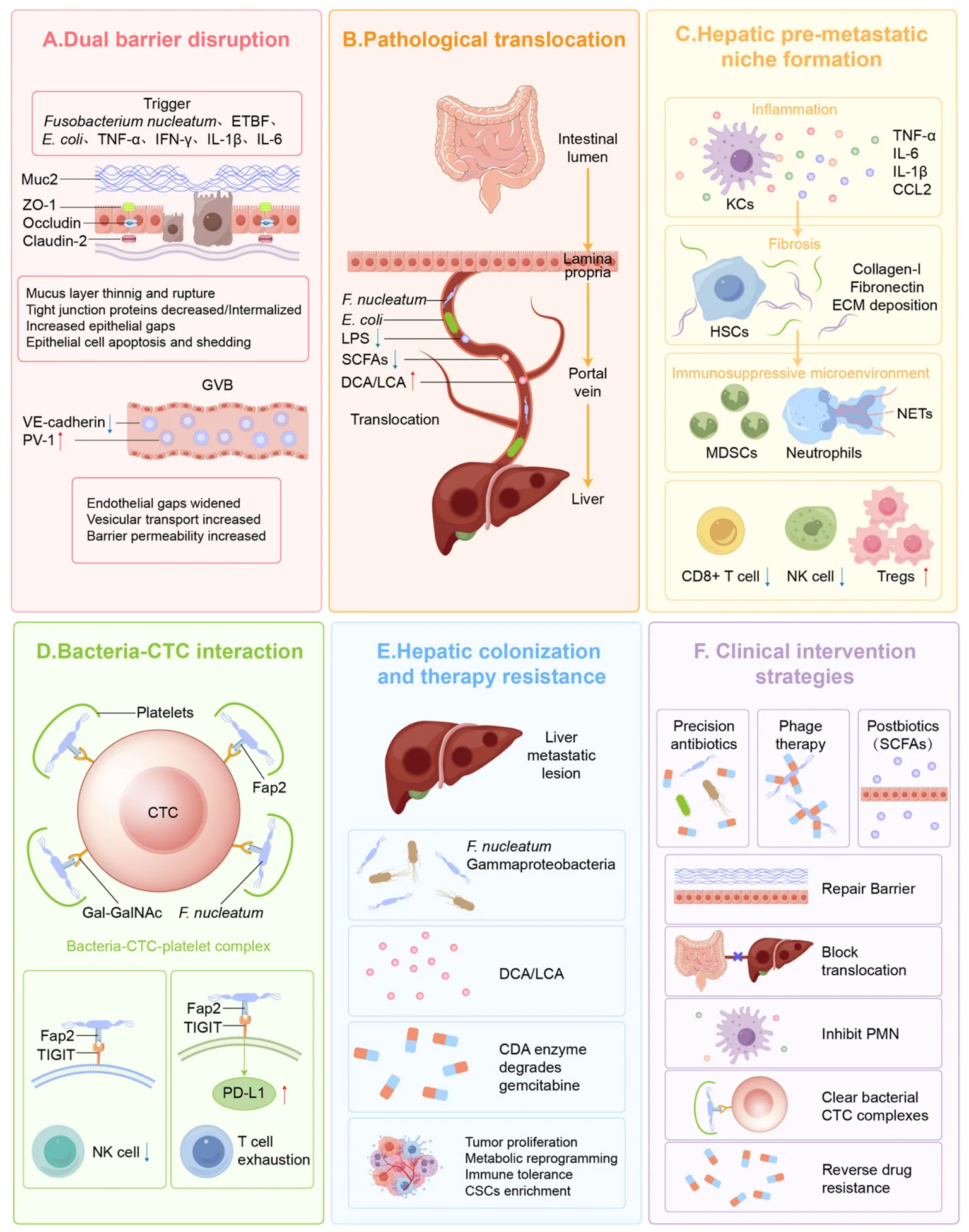
Gut–liver axis drives colorectal cancer liver metastasis. (**A**) Dual barrier disruption: Concurrent impairment of the intestinal epithelial barrier and gut vascular barrier, leading to increased intestinal permeability [12,13,14,15]. (**B**) Pathological translocation: Gut-derived bacteria, lipopolysaccharide (LPS) and metabolites translocate via the portal vein to the liver [12,16,17]. (**C**) Hepatic pre-metastatic niche formation: Kupffer cell activation, hepatic stellate cell-mediated fibrosis, and immune cell remodeling construct an immunosuppressive pre-metastatic niche [15,18,19,20,21,22]. (**D**) Bacteria-CTC interaction: *Fusobacterium nucleatum* adheres to circulating tumor cells (CTCs), promotes platelet coating, and mediates immune escape from natural killer (NK) cell killing [23,24]. (**E**) Hepatic colonization and therapy resistance: Intratumoral microbiota and bile acid remodeling drive tumor cell proliferation, immune tolerance, and chemoresistance in the liver [16,17,25,26]. (**F**) Clinical intervention strategies: Microbiota-oriented therapeutic strategies targeting gut–liver axis to interfere with colorectal cancer liver metastasis progression [27,28]. Blue downward arrows (↓) indicate downregulation of marker expression or decreased cell abundance; red upward arrows (↑) indicate upregulation of marker expression or increased cell abundance. Orange arrows denote microbial translocation, signaling cascades, or directional transport processes; black lines indicate structural labels or general mechanistic connections. Distinct background colors in panels (**A**–**F**) distinguish different stages of the metastatic cascade, and different cell shapes and colors represent distinct cell types as labeled within each panel. All figures were prepared and edited using Adobe Illustrator 2024 (version 28.0).

Although previous reviews have extensively discussed the relationship between gut microbiota and colorectal cancer development, most have focused on tumor initiation, immune regulation, or general therapeutic implications. The specific contribution of gut-derived microbial signals to the multistep process of liver-specific metastatic evolution remains insufficiently characterized. Therefore, this review focuses specifically on the role of the gut–liver axis in CRLM, emphasizing the sequential processes from gut microbiota dysbiosis and intestinal barrier disruption to microbial translocation, hepatic pre-metastatic niche formation, tumor cell–microbiota interactions, and metastatic microenvironment maintenance (Figure 1). Furthermore, we discuss emerging microbiome-based therapeutic strategies and current challenges, including limitations in causal validation, low-biomass microbiome analysis, human clinical evidence, and translational application. By focusing on the metastatic cascade rather than general colorectal carcinogenesis, this review aims to provide a mechanistic framework for understanding how gut-derived microbial signals contribute to CRLM development and may facilitate future precision prevention and treatment strategies.

## 2. Barrier Disruption and Pathological Microbial Translocation

**Figure 2:**
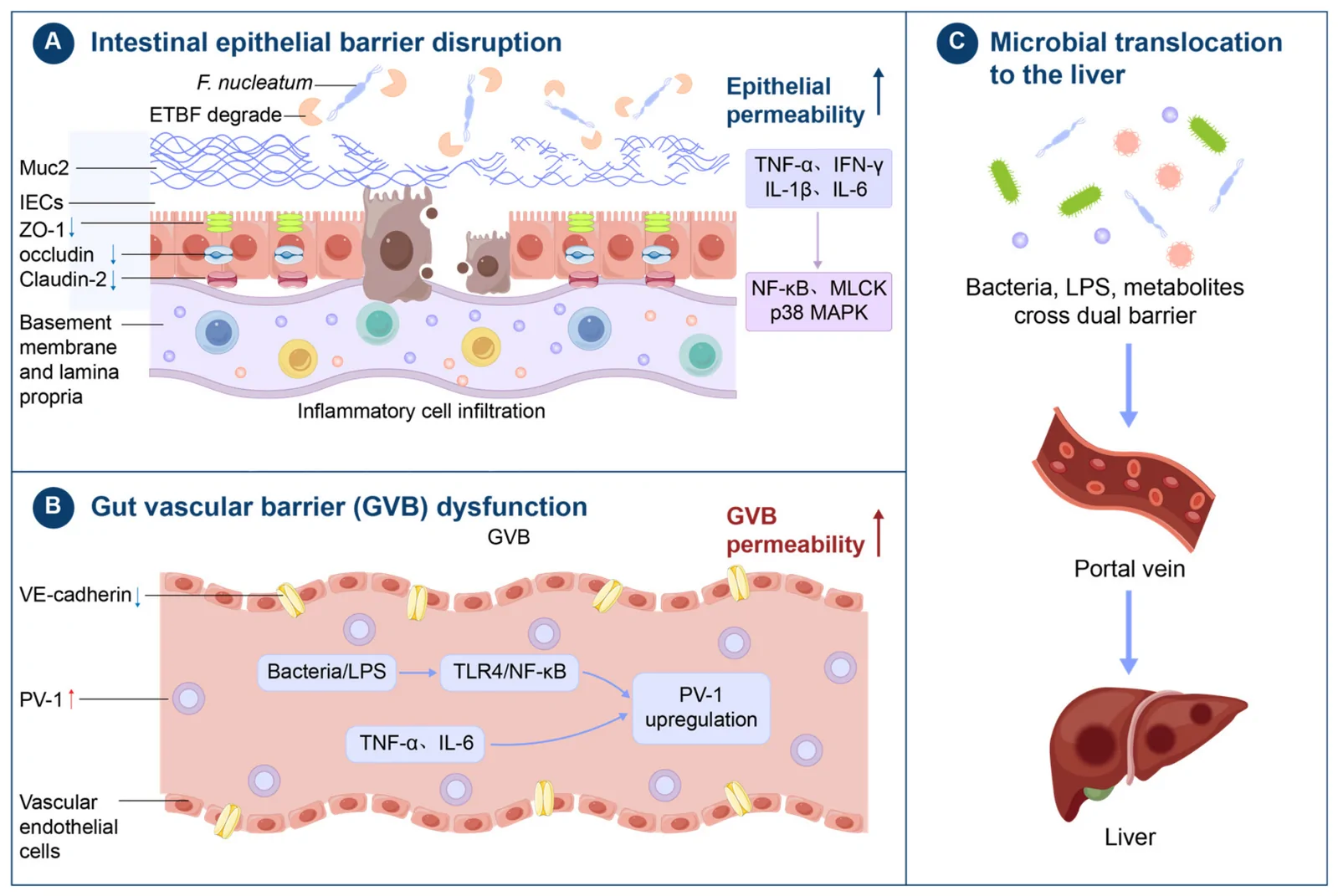
Intestinal epithelial and vascular barrier damage. (**A**) Intestinal epithelial barrier disruption: *Fusobacterium nucleatum* degrades the mucus layer, and pro-inflammatory cytokines downregulate tight junction proteins (ZO-1, occludin), increasing epithelial permeability [12,13,14]. (**B**) Gut vascular barrier (GVB) dysfunction: Bacteria/LPS activate the TLR4/NF-κB pathway, upregulating PV-1 and downregulating VE-cadherin to impair endothelial integrity [12,15,18]. (**C**) Microbial translocation to the liver: Dual barrier injury allows bacteria, LPS and metabolites to enter the portal vein and reach the liver [12,16]. Blue downward arrows (↓) indicate downregulation of tight junction or endothelial junction proteins; red upward arrows (↑) indicate upregulation of protein expression or increased barrier permeability. Blue arrows denote signaling pathway activation or directional transport of microbial components; purple arrows indicate inflammatory cytokine release and downstream signaling cascades. Panels (**A**–*C*) are organized to distinguish different pathological processes, and different cell shapes and colors represent distinct cell types or molecules as labeled within each panel. All figures were prepared and edited using Adobe Illustrator 2024 (version 28.0).

In the multi-stage cascade of colorectal cancer liver metastasis (CRLM), gut dysbiosis accompanied by disturbed microbial metabolism acts as an upstream initiating driver. It triggers sequential impairment of multi-layered intestinal barriers and finally leads to pathological microbial translocation through the portal vein. The intestinal barrier constitutes the essential structural prerequisite for this pathological process, its intact function restrains microorganisms, pathogen-associated molecular patterns, and toxic metabolites from accessing systemic circulation. In the context of CRLM progression, tumor-related inflammation, dysregulated microbiota and metabolic perturbations jointly damage intestinal barrier competence, enabling bacteria and microbial-derived metabolites to cross mucosal barriers and disseminate toward the liver via portal venous circulation. (Figure 2) This cascade represents the starting event of the whole CRLM metastatic sequence, supplying gut-derived pathogenic signals for subsequent hepatic pre-metastatic niche priming [12,29].

Intestinal barrier impairment in CRLM arises downstream of dysbiosis and metabolic disturbances instead of occurring as isolated structural lesions. It proceeds as a continuous pathological cascade across three interconnected layers: the intestinal epithelial barrier, mucus barrier, and intestinal vascular barrier. Disruption of epithelial tight junctions raises paracellular permeability; compromised mucus barriers permit pathogenic bacteria to approach and invade epithelial cells. Furthermore, augmented permeability of the intestinal vascular barrier permits gut-borne microbial signals to pour into portal-venous circulation. These pathological changes reciprocally amplify one another, converting the gut from a defensive boundary confining microbial escape into a major source of gut-derived inflammatory signals delivered to the liver.

### 2.1. Disruption of Intestinal Epithelial Barrier Integrity

Triggered by gut dysbiosis and perturbed microbial metabolism, the intestinal epithelial barrier frequently sustains functional damage in CRLM contexts. Composed of a monolayer of intestinal epithelial cells, intercellular tight-junction complexes and overlying mucus layer, it forms the primary physical defense preventing luminal contents from translocating into host tissues (Figure 2A). Normal epithelial barrier homeostasis depends on balanced microbiota, intact mucus architecture, sufficient epithelial energy metabolism, plus proper expression and subcellular localization of tight-junction proteins [13]. Under CRLM-relevant pathological settings, dysregulated microbiota, inflammatory mediator release and pathogenic bacterial invasion act in concert to disrupt epithelial competence and raise paracellular permeability. Of note, increased intestinal permeability can emerge even at early phases of CRC, implying that barrier dysfunction precedes advanced tumor burden and contributes to early reshaping of tumor-associated microenvironment [14].

#### 2.1.1. Dysbiosis and Epithelial Energy Metabolism Disorders

Under normal circumstances, intestinal commensal bacteria, particularly butyrate-producing bacteria such as *Faecalibacterium prausnitzii*, generate short-chain fatty acids through the fermentation of dietary fiber, providing a crucial energy source for colonic epithelial cells and contributing to the maintenance of mucosal immune homeostasis and barrier integrity. Short-chain fatty acids not only promote epithelial cell energy metabolism but also maintain barrier function by modulating inflammatory responses, enhancing tight junction protein expression, and facilitating mucosal repair.

In CRC patients, the gut microbiota structure often undergoes significant changes, characterized by a decrease in butyrate-producing bacteria and an enrichment of potentially pathogenic bacteria. The decline in butyrate-producing bacteria can lead to insufficient energy supply to epithelial cells, thereby affecting cell renewal, damage repair, and anti-inflammatory capacity. Simultaneously, the reduction in short-chain fatty acids may weaken their inhibitory effect on local inflammatory responses, rendering the epithelial barrier more susceptible to sustained damage from inflammatory factors and bacterial products [14,30]. Exogenous factors such as a high-fat diet can further exacerbate microbial imbalance, promote the expansion of pro-inflammatory or metastasis-associated microbial communities, and establish a positive feedback loop of microbial dysbiosis, barrier damage, and inflammation amplification [31]. Therefore, alterations in microbial metabolic function may serve as a critical link between intestinal microecological imbalance and barrier dysfunction.

In contrast to patients with non-metastatic primary CRC, patients with CRLM exhibit further reshaping of gut microbial community composition: pro-metastatic pathobionts within the genus *Fusobacterium* are enriched, while butyrate-producing commensals exemplified by *Faecalibacterium* undergo further depletion [32]. Such community shifts indicate more profound gut dysbiosis in metastatic disease relative to localized primary CRC. Apart from known depletion of protective short-chain fatty acids, the expanded pathobionts can release virulence-associated proteins and proteases, which may further exacerbate epithelial-mucus barrier injury and amplify intestinal mucosal impairment.

In addition to depleted short-chain-fatty-acid-producing commensals, patients with CRLM also exhibit depleted gut bacteria capable of bile-salt-hydrolase (BSH) activity [16]. Such BSH-deficient microbial configuration perturbs intestinal bile-acid biotransformation, resulting in elevated circulating conjugated primary bile acids (CPBAs) alongside reduced unconjugated bile-acid pools; this metabolic phenotype has been observed in human CRLM cohorts and reproduced in pre-clinical mouse models bearing BSH-deficient gut microbiota [16]. Notably, mechanistic readouts from this study mainly illustrate the prometastatic function of CPBAs within the hepatic microenvironment. Although not directly experimentally validated in this work, altered bile-acid profiles may cooperate with butyrate scarcity and bacterial virulence factors to amplify intestinal barrier vulnerability and further promote pathological microbial translocation across the portal circulation. Accordingly, associations observed in human cohorts remain correlative, and direct causal evidence demonstrating that excess CPBAs disrupt human intestinal barrier integrity awaits further experimental validation [16].

Existing studies also suggest that regulating microbial community structure and restoring short-chain fatty acid levels may help improve barrier function. For instance, probiotic-derived postbiotic preparations can, to some extent, improve microbial community composition and enhance barrier integrity [30]; some traditional Chinese medicine formulations have also been reported to protect the intestinal barrier by modulating the gut microbiota [33]. However, whether these intervention strategies can further reduce the risk of CRC liver metastasis remains to be validated through more mechanistic research and prospective clinical evidence.

#### 2.1.2. Disruption of Tight Junctions Mediated by Inflammatory Factors

The tight junction complex serves as the core structure for maintaining the selective permeability of the intestinal epithelium, comprising proteins such as ZO-1, occludin, and members of the claudin family. The expression levels, subcellular localization, and protein stability of these components are precisely regulated by inflammatory signals [34]. Within the inflammatory microenvironment associated with CRC, tumor cells, immune cells, and microbial components collectively induce the release of pro-inflammatory factors such as TNF-α, IFN-γ, and IL-6, thereby affecting the expression and structural integrity of tight junction proteins and increasing paracellular permeability of epithelial cells [35].

ZO-1, functioning as a crucial scaffold protein that links transmembrane tight junction proteins to the cytoskeleton, plays a pivotal role in epithelial damage repair and the maintenance of junctional structures. A decrease in its expression or abnormal localization can impair mucosal regeneration capacity and prolong barrier damage [34]. Reductions in occludin and claudin-5 can directly disrupt tight junction structures, while an abnormal increase in claudin-2 is associated with heightened epithelial permeability, enhanced tumor invasion, and phenotypes related to liver metastasis [36,37]. Furthermore, molecules such as DDR1 and TRAP1 have been reported to promote tight junction disassembly and epithelial cell damage by regulating signaling axes involving NF-κB, MLCK, and p-MLC2, thereby exacerbating barrier dysfunction [35,36].

It is important to note that a bidirectional relationship may exist between alterations in tight junctions and CRC liver metastasis. On one hand, disruption of tight junctions can facilitate the translocation of bacterial products and induce inflammatory remodeling in the liver; on the other hand, tumor progression itself can further exacerbate barrier damage through inflammatory factors, metabolic stress, and tissue structural disruption. Therefore, when interpreting the relationship between tight junction abnormalities and the risk of liver metastasis, it is essential to avoid simplistic linear causal inferences and instead consider it as a critical component within the intricate interplay network of tumor, inflammation, and the microbiome.

#### 2.1.3. Destructive Effects of Pathogenic Bacteria on the Mucus Barrier

The mucus layer serves as a crucial physical barrier that isolates the gut microbiota from epithelial cells. It is primarily composed of mucins such as MUC2, which restrict direct bacterial contact with epithelial cells and reduce pathogen adhesion and invasion [38]. During the onset and progression of CRC, certain pathogenic or opportunistic bacteria can disrupt the mucus layer structure by secreting proteases, glycosidases, and toxins, thereby compromising its barrier function and exposing epithelial cells more readily to microbial and metabolic stimuli [39].

*F. nucleatum* is a pathogenic bacterium of significant interest in CRC microecology research. Current studies indicate that *F. nucleatum* can promote mucosal barrier damage through epithelial cell adhesion, alteration of mucus glycosylation patterns, and induction of inflammatory responses, potentially contributing to tumor invasion and liver metastasis [39,40]. Furthermore, *F. nucleatum* can enhance tumor cell invasiveness and undermine local barrier defenses by modulating miRNAs, inflammatory cytokines, and epithelial–mesenchymal transition-related pathways [41]. Enterotoxigenic *Bacteroides fragilis* can activate pathways such as STAT3 and ZEB2 by secreting *Bacteroides fragilis* toxin, promoting inflammatory responses and epithelial structural damage, thereby participating in tumor progression [42].

Beyond their well-documented alterations in primary CRC, these pro-metastatic pathobionts display further enrichment in fecal samples from patients with CRLM [32]. Apart from directly degrading mucus layers via virulence-associated effectors, expanded pathobionts within this dysbiotic milieu may also interfere with gut microbial metabolic homeostasis, including perturbing short-chain fatty acid production and reshaping bile-acid profiles. This creates a self-amplifying positive-feedback circuit: pathobiont overgrowth disturbs key microbial metabolic pathways, and resultant metabolic perturbations in turn aggravate epithelial-mucus barrier impairment, which facilitates further outgrowth of harmful taxa.

Microbiome analysis of CRC liver metastasis has revealed that some metastases exhibit a microbial composition similar to that of the primary tumor, suggesting that tumor-associated microbiota may migrate alongside tumor cells and persist in distant lesions [43]. However, liver metastasis represents low-biomass samples, and microbial detection results are susceptible to contamination, sequencing batch effects, and sampling methods. Therefore, these findings necessitate further validation through in situ hybridization, spatial omics, and rigorous negative controls. Overall, mucus barrier disruption may facilitate abnormal activation of the gut–liver axis by enhancing microbial-epithelial interactions, amplifying local inflammation, and promoting microbial translocation.

### 2.2. Alterations in Intestinal Vascular Barrier Permeability

In addition to the epithelial and mucus barriers, the intestinal vascular barrier is a critical defense mechanism that restricts the entry of intestinal microbiota and their products into the systemic circulation (Figure 2B). Comprising intestinal microvascular endothelial cells, intercellular junctions, the basement membrane, and pericytes, this intestinal vascular barrier prevents the direct entry of bacteria and macromolecular toxic substances into the portal vein system to a certain extent [44]. When compromised, intestinal cavity-derived bacteria, lipopolysaccharides, bacterial DNA, and metabolites can more readily traverse the vascular endothelium and reach the liver, triggering innate immune responses and pre-metastatic microenvironment remodeling [12]. In recent years, the role of the intestinal vascular barrier in tumor metastasis has garnered increasing attention. Contrary to the traditional view that the intestinal barrier is primarily determined by epithelial cells, the integrity of the vascular endothelial barrier may govern the efficient entry of intestinal-derived signals into the portal vein circulation [45]. Therefore, abnormalities in the intestinal vascular barrier may serve as a crucial link between local intestinal lesions and distant hepatic microenvironment alterations.

#### 2.2.1. Upregulation of PV-1 Expression in Endothelial Cells Induced by Bacterial Signals 

PV-1 is a crucial protein that regulates vascular endothelial permeability, and its increased expression typically indicates compromised endothelial barrier function. Studies have demonstrated that intestinal pathogens and their associated signals can upregulate PV-1 expression by influencing the Wnt/β-catenin and other pathways in endothelial cells, thereby enhancing vascular endothelial permeability [46]. For instance, specific strains of *Escherichia coli* can inhibit Wnt/β-catenin signaling and induce an elevation in PV-1 levels, consequently impairing the integrity of the intestinal vascular barrier [46].

Beyond direct stimulation by pathogenic bacterial strains, inflammatory mediators derived from the dysbiotic gut milieu, such as lipopolysaccharide and aberrant bile-acid species, may also contribute to PV-1 upregulation in intestinal endothelial cells via creating a local pro-inflammatory microenvironment [47]. This expands the conceptual framework: gut–microbiota-dysbiosis-related metabolic signals, in addition to bacterial virulence factors, can compromise gut-vascular barrier permeability, forming a complementary regulatory axis parallel to epithelial-mucus barrier injury described in previous sections.

Additionally, intestinal vascular-associated macrophages may also participate in the regulation of the vascular barrier. When these cells are abnormally activated, they can further amplify the effects related to PV-1 and promote increased vascular permeability by releasing inflammatory factors and modulating endothelial cell function [45]. In the context of tumors, tumor-derived exosomes, inflammatory mediators, and microbial products may collectively act on the intestinal vascular endothelium, gradually diminishing the vascular barrier’s ability to restrict intestinal-derived signals [12].

From a clinical translation perspective, intestinal permeability, circulating bacterial DNA, Lipopolysaccharide (LPS) levels, and related endothelial damage indicators may provide an indirect basis for evaluating intestinal vascular barrier function [44]. However, there remains a lack of a standardized detection system capable of directly and dynamically reflecting the intestinal vascular barrier status in CRC patients, which represents a significant challenge to be addressed in the future.

#### 2.2.2. Vascular Barrier Disruption and Liver-Directed Translocation

Once the intestinal vascular barrier is compromised, enterogenous bacteria and their products can more readily enter the portal vein circulation and reach the liver via blood flow. Given that the liver is the first organ encountered in portal venous reflux, Kupffer cells, hepatic sinusoidal endothelial cells, and other innate immune cells are continuously exposed to intestinal microbial signals. This persistent stimulation can induce low-grade liver inflammation, myeloid cell recruitment, enhanced immune tolerance, and matrix remodeling, thereby creating a more favorable microenvironment for the adhesion, retention, and colonization of subsequent CTCs [12]. Previous studies have suggested a correlation between intestinal vascular barrier damage and an increased risk of bacterial dissemination and CRC liver metastasis [12]. Concurrently, some intervention studies have indicated that restoring vascular barrier function may help reduce bacterial translocation and inflammatory damage to distant organs. For example, terlipressin has been reported to mitigate bacterial translocation-related damage by improving vascular barrier function [44]; certain traditional Chinese medicine compounds also exhibit potential in maintaining vascular barrier integrity and reducing liver injury [45]. However, these findings are predominantly derived from animal experiments or specific disease models, and their direct applicability to the prevention of CRC liver metastasis requires further validation.

Collectively, damage across multi-layer intestinal barriers furnishes the structural prerequisite for gut-derived microorganisms and their metabolites to access the liver, representing an early initiating event for pathological gut–liver axis activation. Synergistic injury to epithelial, mucus and intestinal vascular barriers persistently amplifies hepatic exposure to portal-vein-borne microbial cues, which further drive inflammatory response, immune cell recruitment and matrix remodeling toward pre-metastatic niche construction (Figure 2C). These gut-originated microbial and metabolic signals delivered via portal circulation will further initiate hepatic immune reprogramming, as elaborated in Section 3. Nevertheless, causal evidence linking intestinal barrier disruption specifically to human CRLM remains incomplete. Future investigations should combine prospective clinical cohorts, dynamic barrier-function readouts, spatial localization approaches and multi-omics profiling to disentangle the temporal sequence and functional weight of barrier impairment during liver-metastasis progression.

Mechanistic investigations performed in ulcerative colitis experimental models have illustrated that botanical natural products are capable of remodeling gut community composition, restoring metabolite homeostasis encompassing short-chain fatty acids, tryptophan-derived signals and bile-acid profiles, and reinforcing mucosal barrier integrity [48]. It should be noted that such experimental readouts are predominantly generated from inflammatory-bowel-disease-oriented pre-clinical systems and cannot be directly extrapolated to colorectal-cancer-associated liver-metastasis contexts.

## 3. Immune Remodeling of Hepatic Pre-Metastatic Niches

**Figure 3:**
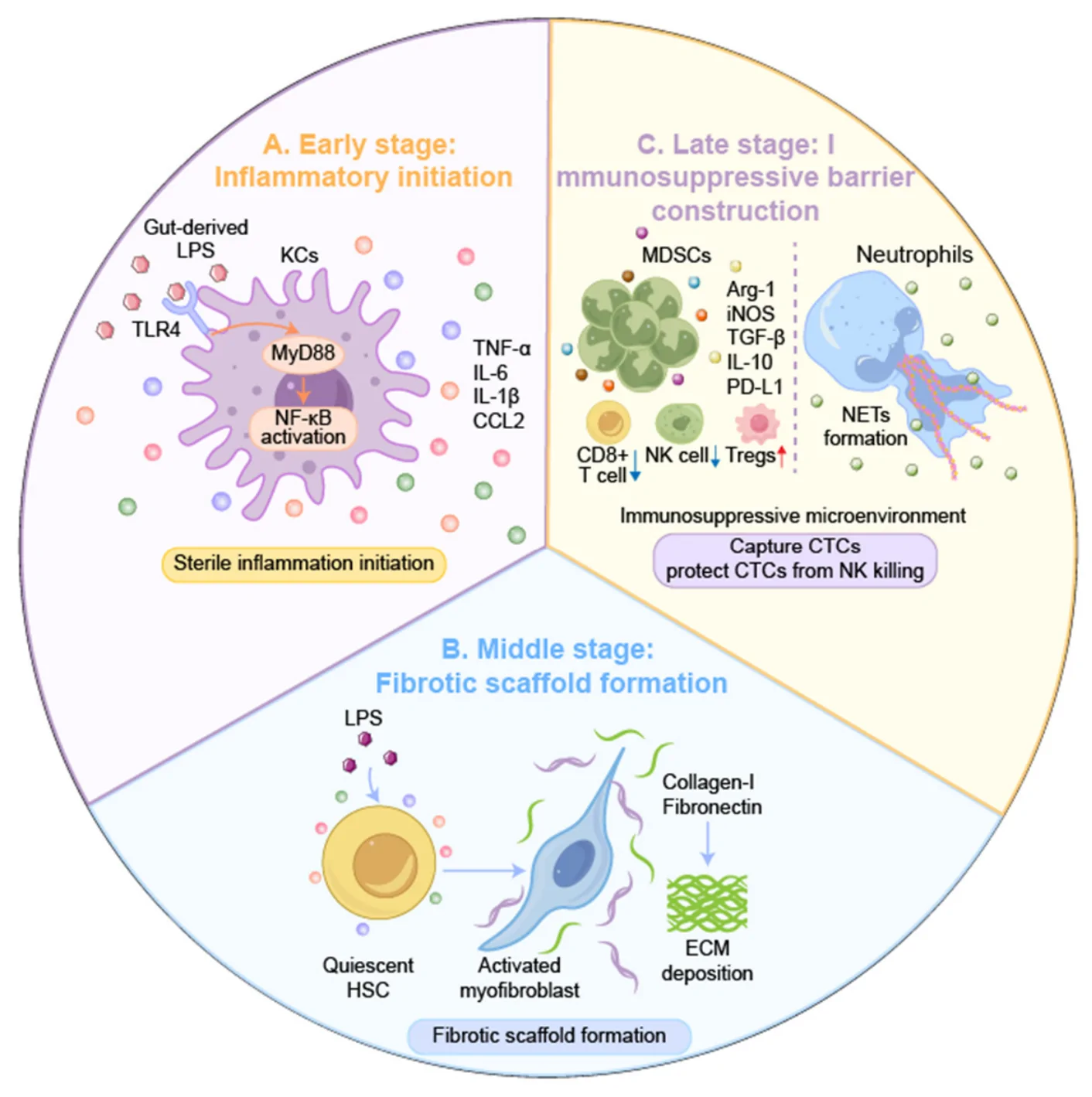
Spatiotemporal reshaping of hepatic pre-metastatic niche. (**A**) Early stage: Inflammatory initiation: Gut-derived LPS activates Kupffer cells via the TLR4/MyD88 pathway, triggering pro-inflammatory cytokine release and sterile inflammation [15,18,19]. (**B**) Middle stage: Fibrotic scaffold formation: LPS and inflammatory signals activate hepatic stellate cells, promoting ECM deposition and constructing a fibrotic matrix [20,21,53,54]. (**C**) Late stage: Immunosuppressive barrier construction: Accumulation of MDSCs and formation of NETs suppress anti-tumor immunity, establishing an immunosuppressive microenvironment [22]. Blue arrows indicate signaling pathway activation or cellular phenotypic transition; purple/pink arrows denote inflammatory cytokine release; dashed arrows with downward arrows (↓) indicate suppression or downregulation of immune cell function. Panels (**A**–**C**) use distinct background colors (light purple, light blue, and light yellow) to distinguish the early, middle, and late stages of pre-metastatic niche formation, respectively. Different cell shapes and colors represent distinct cell types or molecules as labeled within each panel. All figures were prepared and edited using Adobe Illustrator 2024 (version 28.0).

Once the intestinal barrier is compromised, intestinal-derived microbial components and their metabolites can continuously infiltrate the liver via the portal vein, subjecting the liver’s innate immune system to prolonged low-grade inflammatory stimulation (Figure 3). Given the liver’s dual functions of immune surveillance and tolerance, its response to intestinal-derived signals extends beyond mere inflammatory clearance, leading to gradual remodeling of immune cell composition, cytokine networks, matrix architecture, and metabolic states under sustained stimulation. This remodeling process may precede the stable colonization of CTCs, creating a conducive microenvironment for their subsequent adhesion, survival, and proliferation, thereby serving as a crucial foundation for the formation of CRC liver pre-metastatic niches. The hepatic pre-metastatic niche is not driven by a single cell type or signaling pathway but represents a dynamic ecological process involving Kupffer cells, liver sinusoidal endothelial cells, hepatic stellate cells, neutrophils, myeloid-derived suppressor cells, natural killer cells, NKT cells, and the extracellular matrix. Its core characteristics encompass pre-activation of liver innate immune cells, release of pro-inflammatory and chemotactic factors, recruitment of myeloid cells, activation of hepatic stellate cells, matrix deposition, enhanced immunosuppression, and weakened anti-tumor immune surveillance. Current studies indicate that these alterations collectively enhance the liver’s capacity to accommodate CRC CTCs; however, many of the underlying mechanisms are primarily derived from animal models and in vitro experiments, with direct evidence from human metastatic stages remaining relatively scarce.

### 3.1. Pre-Activation of Innate Immune Cells

#### 3.1.1. Recognition and Response of Kupffer Cells to Intestinal-Derived Signals

As resident hepatic macrophages, Kupffer cells act as key downstream effector cells responding to gut–microbiota-derived signals translocated via the portal vein after intestinal barrier disruption [15,18]. Upon gut barrier impairment, pathogen-associated molecular patterns such as lipopolysaccharide, peptidoglycan, and bacterial DNA travel through portal venous circulation to the hepatic sinusoids and are sensed by pattern-recognition receptors expressed on Kupffer-cell surfaces. Notably, TLR4-mediated LPS recognition represents a well-characterized pathway [15,18]. LPS binding to TLR4 activates NF-κB and other inflammatory transcription factors through the MyD88-dependent signaling pathway, promoting the transition of Kupffer cells from a steady-state surveillance phenotype to a pro-inflammatory activated state [18]. This process induces the release of inflammatory cytokines and chemokines, including TNF-α, IL-6, IL-1β, and CCL2, thereby amplifying local inflammatory responses and providing a signaling basis for subsequent myeloid cell recruitment and liver microenvironment remodeling [49]. Consequently, Kupffer cells serve as a pivotal link between intestinal microbial translocation and immune remodeling in the liver pre-metastatic niche. (Figure 3A).

Of note, Kupffer-cell functions are stage-dependent. Upon persistent stimulation, these cells can shift between pro-inflammatory and immunosuppressive states under the combined action of gut–microbiota-derived stimuli and secondary tumor-derived factors.

#### 3.1.2. Intestinal-Derived PAMPs-Mediated Low-Grade Chronic Inflammation

Intestinal-derived microbial products, upon continuously entering the liver, can induce a low-grade chronic inflammatory state characterized by innate immune activation and cytokine release. Unlike typical infectious inflammation, this process does not necessarily involve persistent infection with viable bacteria but is more prominently manifested as immune activation and microenvironment remodeling under the continuous stimulation of PAMPs. Therefore, a more accurate description than simply defining it as aseptic inflammation would be a non-infectious inflammatory response driven by intestinal microbial signals.

Activated Kupffer cells release pro-inflammatory cytokines such as TNF-α, IL-6 and IL-1β, and recruit circulating monocytes, neutrophils, and immature myeloid cells into the liver through chemokines like CCL2 [19,49,50]. Among these, IL-6 not only amplifies the inflammatory response but also influences tumor cell survival, hepatocyte stress responses, and liver microenvironment plasticity through signaling pathways such as JAK/STAT [50]. CCL2-mediated monocyte recruitment provides a cellular basis for the expansion of liver myeloid cells and the formation of immunosuppressive networks [19]. The critical significance of this chronic low-grade inflammation lies in its potential to alter the liver’s response to subsequent CTCs, rather than merely being a local immune reaction. On one hand, inflammatory factors enhance the expression of endothelial adhesion molecules in hepatic sinuses, promoting the retention of CTCs; on the other hand, persistent inflammation may induce immune tolerance and infiltration of myeloid-derived suppressor cells, gradually creating a microenvironment more conducive to tumor cell survival.

#### 3.1.3. Imbalance of Kupffer Cell Function and Weakening of Immune Surveillance

Under the continuous influence of LPS and other intestinal-derived stimuli, Kupffer cells may undergo functional reprogramming, characterized by decreased phagocytic activity, altered antigen-presentation capacity, sustained release of inflammatory factors, and enhanced immune regulatory functions [51]. This state may impair the liver’s ability to clear CTCs entering the hepatic sinuses at an early stage, providing opportunities for tumor cells to temporarily reside and subsequently colonize. However, direct human evidence documenting such Kupffer-cell functional reprogramming within the pre-metastatic liver remains limited and awaits further investigation. Therefore, the role of Kupffer cells in CRLM should be understood as a dynamic process: initially, they may initiate an inflammatory response by recognizing intestinal-derived signals; later, under the influence of continuous stimulation and tumor-derived signals, they may shift towards immunosuppression and support for metastasis.

#### 3.1.4. Heterogeneity and Research Limitations of Liver Macrophage Subsets

Liver macrophages encompass heterogeneous subsets including tissue-resident Kupffer cells and monocyte-derived macrophages [19]. These subsets differ in their responsiveness to portal-translocated gut–microbiota-derived stimuli. At present, most CRLM pre-metastatic-niche studies do not sufficiently distinguish these subsets. Single-cell and spatial transcriptomic approaches are therefore required to dissect their respective roles downstream of gut–microbiota-derived signals.

### 3.2. Construction of a Liver Fibrosis-like Microenvironment

#### 3.2.1. Activation and Phenotypic Transformation of Hepatic Stellate Cells

Acting as downstream stromal effector cells, hepatic stellate cells mediate extracellular-matrix remodeling and fibrotic responses triggered by gut–microbiota-originated inflammatory cascades transmitted via portal-venous circulation [20,21]. Under homeostatic conditions, hepatic stellate cells predominantly remain in a quiescent state, storing vitamin A and maintaining the stability of the hepatic sinusoidal microenvironment. Following intestinal-barrier disruption, portal-translocated microbial stimuli provoke Kupffer-cell-derived release of TNF-α, IL-6, and TGF-β; together with tumor-derived factors, these mediators drive gradual hepatic stellate-cell activation, characterized by a reduction in lipid droplets, enhanced proliferation, increased α-SMA expression, and the formation of a myofibroblast-like phenotype [20,21]. Activated hepatic stellate cells synthesize and secrete a substantial amount of extracellular matrix components, including type I collagen, fibronectin, and laminin, among others (Figure 3B), leading to perisinusoidal matrix deposition and an increase in local tissue stiffness [21]. These alterations provide more favorable adhesion and survival scaffolds for CTCs and may also influence tumor cell invasiveness by altering mechanical signal transduction.

Beyond gut–microbiota-driven inflammatory inputs, autophagy-related pathways can modulate hepatic stellate-cell activation [52]. Nevertheless, whether hepatic stellate-cell activation represents an essential prerequisite for CRC pre-metastatic niche formation remains incompletely clarified.

#### 3.2.2. Excessive Extracellular Matrix Deposition and Matrix Sclerosis

Extracellular matrix deposition constitutes a crucial structural foundation for the formation of the pre-metastatic microenvironment in the liver. Activated hepatic stellate cells and myofibroblast-like cells can modify the local matrix composition and mechanical properties of the liver by secreting collagen, fibronectin, and other matrix proteins [21]. As ECM deposition increases, liver tissue stiffness rises, and the structure and cellular arrangement of hepatic sinuses may undergo alterations, thereby affecting the retention and adhesion of CTCs in the liver. Matrix sclerosis not only provides physical support but also influences the functions of tumor cells and stromal cells through mechanical signal transduction. For instance, increased matrix stiffness can activate YAP/TAZ, FAK, E2F3, and related mechanosensitive signaling pathways, promoting tumor cell survival, migration, and proliferation, and further maintaining the activated state of hepatic stellate cells. The positive feedback loop between mechanical signals and matrix deposition may drive the pre-metastatic microenvironment towards a state more conducive to tumor colonization. Several non-coding-RNA-dependent regulatory axes can also modulate ECM-related hepatic stellate-cell activity [53,54]; however, their specific roles within the gut–microbiota-triggered pre-metastatic cascade in human CRLM remain poorly defined.

#### 3.2.3. Supportive Role of Fibrosis-like Matrix in Tumor Colonization

Fibrosis-like matrix is not merely a consequence of tissue repair but may participate in pre-metastatic niche formation through multiple mechanisms. Firstly, the increased deposition of ECM provides adhesion sites for CTCs, facilitating their retention in liver sinuses or parenchyma. Secondly, matrix components such as fibronectin and collagen enhance tumor cell anti-apoptotic and migratory capabilities via integrin signaling. Thirdly, increased matrix stiffness activates mechanotransduction pathways in tumor cells, improving their adaptability to the liver microenvironment. Finally, the dense matrix may alter immune cell infiltration and spatial distribution, indirectly weakening local anti-tumor immune responses [54].

Thus, liver fibrosis-like alterations serve as a crucial intermediary between inflammatory responses and immunosuppression. They result from persistent intestinal inflammation and tumor-derived signals while further promoting tumor cell colonization and growth. Future research should elucidate the relationships between liver fibrosis severity, matrix stiffness, and liver metastasis risk in CRC patients, and assess the potential of anti-fibrotic interventions in preventing or delaying CRLM.

### 3.3. Establishment of the Immunosuppressive Barrier

#### 3.3.1. Recruitment and Immunosuppressive Functions of MDSCs

Myeloid-derived suppressor cells (MDSCs) represent important downstream immunosuppressive effector cells recruited to the pre-metastatic liver in response to gut–microbiota-derived inflammatory stimuli after intestinal barrier impairment, and can be classified into monocytic MDSCs (Mo-MDSCs) and granulocytic MDSCs (G-MDSCs) (Figure 3C). Both intestinal-barrier-derived inflammatory signals and tumor-derived factors recruit MDSCs from the bone marrow and peripheral blood to pre-metastatic liver regions via signaling axes involving CCL2, CCL7, CXCL1, CXCL2, and S1PR1-STAT3. Upon entering the liver, MDSCs suppress anti-tumor immune responses through multiple mechanisms. Firstly, they express arginase 1 and inducible nitric oxide synthase (iNOS), depleting arginine and producing nitric oxide to inhibit CD8+ T cell proliferation and cytotoxicity. Secondly, they secrete immunosuppressive cytokines such as IL-10 and TGF-β, promoting T cell exhaustion and immune tolerance. Thirdly, MDSCs inhibit NK cell activity and induce macrophages and T cells to adopt immunosuppressive phenotypes [55]. Some studies further suggest that CCL7 secreted by Mo-MDSCs maintains hepatic immunosuppression and facilitates tumor cell dormancy and subsequent growth [56]. Signaling pathways including NLRP6, USP15, and SMYD3-CCL2 have also been implicated in MDSC infiltration and immune evasion [57,58]. However, MDSCs exhibit significant heterogeneity, with different subpopulations playing distinct roles during the pre-metastatic, colonization, and growth phases of CRLM. Therefore, future studies should employ single-cell sequencing and functional assays to dissect their subpopulation-specific characteristics and stage-dependent functions.

#### 3.3.2. Neutrophil Extracellular Traps and the Metastasis-Promoting Microenvironment

Neutrophil extracellular traps (NETs) represent one downstream pathological output triggered by portal-vein-delivered gut–microbiota-derived danger signals after intestinal barrier disruption. These web-like structures, mainly composed of DNA, histones and granular proteins, are produced when neutrophils undergo NETosis. LPS and inflammatory cytokines translocated via portal circulation, together with tumor-derived signals, can induce neutrophil NETosis and drive NET formation within the hepatic pre-metastatic niche [22]. NETs may contribute to CRC liver metastasis through two primary mechanisms. On one hand, the web-like structure of NETs can capture CTCs, facilitating their retention and adhesion within the hepatic sinuses. On the other hand, NETs can alter the local immune microenvironment, impairing the recognition and killing of tumor cells by NK cells and CD8+ T cells, thereby enhancing the tumor cells’ ability to evade immune surveillance [22]. Additionally, NETs may promote ECM remodeling, endothelial adhesion molecule expression, and tumor metabolic adaptation by releasing proteases, histones, and inflammatory mediators.

In recent years, several molecules have been implicated in NET formation and their metastasis-promoting effects. For instance, PRIM1 can enhance neutrophil recruitment and NET generation [59]. Granulocytic myeloid-derived suppressor cells (MDSCs) can synergize with neutrophils to amplify the metastasis-promoting effects associated with NETs [60]. NETs can also influence the expression of metabolism-related molecules such as LDHA, promoting metabolic reprogramming in tumor cells [61]. Signals related to SPP1 are also believed to participate in NET formation and the regulation of the liver metastasis microenvironment [62]. However, the dynamic changes, specific biomarkers, and therapeutic intervenability of NETs in human CRLM still require validation with more clinical samples.

#### 3.3.3. NK Cells, NKT Cells, and Weakened Anti-Tumor Immunity

NK and NKT cells constitute key hepatic anti-tumor surveillance populations whose functional impairment represents a downstream consequence of sustained portal-borne gut–microbiota-derived inflammatory stimulation. The liver is rich in NK cells and NKT cells, which play crucial roles in eliminating abnormal cells and maintaining tumor immune surveillance. Under physiological conditions, NK cells can directly kill tumor cells via the perforin and granzyme pathway, while NKT cells participate in anti-tumor responses by rapidly secreting cytokines and modulating adaptive immunity. Nevertheless, persistent portal-translocated intestinal inflammatory stimuli, together with myeloid suppressor-cell infiltration and metabolic reprogramming, can jointly suppress the anti-tumor function of these effector lymphocytes.

MDSCs, Treg cells, immunosuppressive macrophages, and tumor-derived metabolites can inhibit the function of NK cells and CD8+ T cells, leading to decreased killing capacity, reduced expression of activation receptors, and increased expression of exhaustion-related molecules [22,55,56,57,58,59,60,61,62,63]. Furthermore, alterations in bile acid metabolism may regulate the recruitment and function of hepatic NKT cells by affecting the CXCL16-CXCR6 axis. It is important to note that the effects of bile acids on liver immunity are dependent on their type, concentration, and the model used, with different bile acid molecules potentially exerting distinct or even opposing immune effects. Therefore, when discussing the relationship between bile acids and NKT cells, one should avoid oversimplifying it into a single pro-metastatic or anti-metastatic mechanism.

Overall, the decline in NK and NKT cell function may serve as an important indicator of the liver’s transition from immune surveillance to immune tolerance. However, whether this process occurs before the formation of liver metastasis in CRC patients and its direct causal relationship with intestinal microbial signals still require further confirmation through prospective clinical samples and spatial immunoassays.

#### 3.3.4. Collaborative Remodeling of Immune, Stromal, and Metabolic Networks

Hepatic pre-metastatic niche formation represents a cascade of downstream pathological events primarily initiated by translocated gut–microbiota-derived microbial signals passing through the portal vein, rather than a process driven purely by intrinsic hepatic immune-cell activity. This multi-step process involves collaborative remodeling among immune, stromal, and metabolic networks. Intestinal-derived microbial signals initially activate Kupffer cells and hepatic sinusoidal endothelial cells, triggering the release of inflammatory factors and chemokines. Subsequently, MDSCs, neutrophils, and monocyte-derived macrophages gradually infiltrate, creating an immunosuppressive environment.

Concurrently, the activation of hepatic stellate cells and ECM deposition alter the local tissue architecture, providing physical support for tumor cell adhesion and survival. Metabolites such as bile acids, lactic acid, and short-chain fatty acids further influence immune cell function and tumor cell adaptability. This networked process suggests that hepatic pre-metastatic niches should not be simply viewed as cellular or molecular abnormalities but rather as dynamic microecosystems formed through the interplay of intestinal-derived signals, host immune responses, stromal remodeling, and tumor-derived factors. Single-cell transcriptomics, spatial transcriptomics, and spatial proteomics technologies have begun to elucidate the dynamic changes in immune cell composition and spatial distribution within the liver metastasis microenvironment [46]. (Table 1) Additionally, exercise interventions, traditional Chinese medicine formulations, and strategies targeting NETs or myeloid cells have been reported to partially reverse immunosuppression and inflammatory remodeling [64,65,66], although their clinical translation still requires standardized models and rigorous validation.

In summary, immune and stromal remodeling within hepatic pre-metastatic niches constitutes one major downstream pathological outcome evoked by translocated gut–microbiota-derived microbial signals reaching the liver via portal-venous circulation during CRLM progression. By activating Kupffer cells, triggering chronic hepatic inflammation, promoting hepatic stellate-cell activation and ECM deposition, recruiting MDSCs and neutrophils, and dampening NK-, NKT- and CD8+-T-cell-mediated anti-tumor immunity, these gut-originated signals collectively establish a liver microenvironment permissive for circulating tumor-cell colonization. However, current evidence primarily stems from animal models and mechanistic studies, with human pre-metastatic niche samples being difficult to obtain. The causal relationship between intestinal microbial signals and liver immune remodeling remains to be further elucidated. Future research should integrate longitudinal clinical cohorts, portal vein blood microbial detection, spatial multi-omics, and functional intervention experiments to systematically dissect the temporal patterns and intervention points in hepatic pre-metastatic niche formation.

## 4. Microbial Regulation of CTCs and Metastatic Colonization in Colorectal Cancer Liver Metastasis

Once tumor cells detach from the primary site of CRC, they gain access to the portal-venous circulation through mesenteric veins before arriving at the liver. Uniquely for colorectal carcinoma, the portal vein creates a direct anatomical conduit linking the intestinal microenvironment with the hepatic vasculature, distinguishing it from most other extra-intestinal solid tumors that metastasize to the liver via systemic arterial circulation. As described in Section 2, impaired intestinal epithelial and gut-vascular barriers permit gut-derived microbes and microbial-derived mediators to spill into this portal-venous bloodstream. These circulating microbial signals can exert dual-layered effects: they not only prime the hepatic pre-metastatic niche as illustrated in Section 3, but also directly interact with circulating tumor cells (CTCs) within portal blood. The circulatory phase therefore represents a critical bottleneck in the tumor-metastasis cascade, during which CTCs must withstand multiple stresses, including blood-flow shear force, anoikis, oxidative stress, immune surveillance, and coagulation-system-mediated regulation.

Traditionally, this circulatory window has been interpreted merely as a passive bloodstream-based selection filter for tumor cells. However, accumulating evidence demonstrates that intestinal microorganisms and their released bioactive molecules can actively modulate CTC survival, immune evasion, and subsequent hepatic colonization potential via crosstalk with tumor-cell, platelet, immune-cell, and exosome-associated networks. In the context of CRC liver metastasis, *Fusobacterium nucleatum* (*F. nucleatum*) stands out as a well-studied representative tumor-associated bacterium (Table 2). This bacterium not only accumulates in the primary CRC site but has also been linked to tumor invasion, immunosuppression, treatment resistance, and poor prognosis [23,24]. Mechanistically, *F. nucleatum* may enhance the adaptability of CTCs within the portal vein system through several pathways: Fap2–Gal-GalNAc-mediated tumor-cell adhesion, formation of bacterial–platelet–tumor-cell complexes, Fap2–TIGIT-mediated inhibition of NK-cell function, lactic-acid-metabolism-related immune modulation, and exosome-mediated amplification of distant signals. It is important to note that while some of these mechanisms are currently supported by robust cell and animal experiments, further direct clinical evidence is needed to confirm whether they occur sequentially in the human portal-vein circulation and ultimately determine the outcome of CRC liver metastasis.

**Table 2:** Key Microorganisms and Their Functional Mechanisms in CRC Liver Metastasis.

Microorganism	Location/Distribution	Key Molecules	Stage of Action	Functional Mechanisms	Reference
*Fusobacterium nucleatum* (Fn)	Gut, primary tumor, liver metastasis, surface of circulating tumor cells (CTCs)	Fap2, LPS, outer membrane proteins, exosomes	Barrier damage, translocation, circulation, colonization, metastasis maintenance	Degrades intestinal mucus layer (Muc2) and increases intestinal permeability; Fap2 binds Gal-GalNAc on CTCs to form bacteria-tumor complexes; Activates platelets to form bacteria-platelet-tumor microemboli, resisting shear stress and promoting survival; Fap2 engages TIGIT on NK cells to inhibit cytotoxicity and mediate immune escape; Induces lactate production and suppresses CD8+ T/NK cells; Promotes liver colonization, proliferation, stemness and chemoresistance	[12,23,24,25,26]
Enterotoxigenic *Bacteroides fragilis* (ETBF)	Gut, primary tumor	BFT (*Bacteroides fragilis* toxin)	Barrier damage	Activates STAT3/ZEB2 signaling via BFT to degrade mucus layer; Disrupts tight junctions, upregulates claudin-2 and increases epithelial permeability; Induces inflammation, promotes EMT and drives tumor progression and liver metastasis	[13,14]
*Escherichia coli* (e.g., NF731)	Gut	LPS, outer membrane proteins	Barrier damage, translocation	Inhibits Wnt/β-catenin signaling, upregulates PV-1 and disrupts gut vascular barrier (GVB); Promotes translocation of bacteria/LPS to liver via portal vein; LPS activates Kupffer cells via TLR4/NF-κB and initiates sterile inflammation	[12,15,18]
Gammaproteobacteria	Liver metastasis, intratumor	CDA (cytidine deaminase)	Metastasis maintenance, therapeutic resistance	Highly expresses CDA to degrade gemcitabine and mediate chemoresistance; Activates NF-κB/MAPK signaling, upregulates drug efflux pumps and promotes stemness	[25,26]
*Enterococcus* spp.	Liver metastasis, intratumor	CDA, exosomes	Metastasis maintenance, therapeutic resistance	Expresses CDA to inactivate gemcitabine; Secretes metabolites/exosomes to induce DNA damage repair and chemoresistance	[25,26]
*Bacteroides* spp. (non-ETBF)	Gut, primary tumor, liver metastasis	LPS, bile salt hydrolase (BSH)	Translocation, metabolic reprogramming	Mediates bile acid dyshomeostasis: reduced BSH → increased primary bile acids (TCA/GCA), decreased secondary bile acids (DCA); DCA regulates CXCL16–CXCR6 axis, inhibits NKT cells and promotes immune tolerance; LPS promotes inflammation, translocation and hepatic pre-metastatic niche (PMN) formation	[16,17]
*Prevotella* spp.	Gut, primary tumor, liver metastasis	LPS, metabolites	Barrier damage, translocation, PMN formation	Enriched during dysbiosis, positively correlated with liver metastasis risk; Pro-inflammatory, enhances intestinal permeability and participates in hepatic immune remodeling	[12,13,14]
Butyrate-producing bacteria (e.g., *Faecalibacterium*)	Gut	Short-chain fatty acids (SCFAs: butyrate, acetate, propionate)	Barrier damage, immune regulation	Synthesizes butyrate for enterocyte energy and maintains tight junctions (ZO-1/occludin); Anti-inflammatory, downregulates TNF-α/IFN-γ; Significantly reduced in liver metastasis patients → barrier impairment and enhanced inflammation	[16]

Notes: CRC, colorectal cancer; Fn, *Fusobacterium nucleatum*; ETBF, enterotoxigenic *Bacteroides fragilis*; CTC, circulating tumor cell; CDA, cytidine deaminase; BSH, bile salt hydrolase; SCFAs, short-chain fatty acids. Microbial functions are categorized according to different stages of the gut–liver axis-mediated metastatic cascade.

### 4.1. Bacterial-Assisted Survival of CTCs

Beyond viable pathobionts physically adhering to circulating colorectal-cancer cells, portal-venous blood also carries soluble gut-derived microbial mediators released upon gut-vascular-barrier disruption [12]. These circulating bioactive substances, including lipopolysaccharides and microbially modified bile-acid species, do not require direct physical contact with tumor cells but can remodel the intravascular immune milieu to alter CTC survival and metastatic competence [16,67]. The following sections first describe mechanisms driven by bacteria physically associated with CTCs, before illustrating how these microbial signals facilitate platelet-dependent protection, coagulation activation, and immune-evasive phenotypes.

#### 4.1.1. Fap2–Gal-GalNAc Mediated Specific Adhesion

As one of the most studied Gram-negative anaerobic bacteria in the microecology associated with CRC, *F. nucleatum* can specifically bind to tumor cells via surface adhesion factors. Notably, the interaction between the Fap2 adhesin and the Gal-GalNAc glycosyl structure on the surface of tumor cells represents a key mechanism underlying the selective enrichment of *F. nucleatum* in CRC tissues [23]. During malignant transformation, CRC cells frequently exhibit abnormal glycosylation modifications, leading to increased expression of glycosyl epitopes such as Gal-GalNAc, whereas such structures are relatively scarce on the surface of normal intestinal epithelial cells. This disparity provides a molecular basis for the selective adhesion of *F. nucleatum* to tumor cells [24].

The Fap2 protein recognizes the Gal-GalNAc glycosyl epitope and facilitates the stable binding of *F. nucleatum* to the surface of CRC cells. This binding not only contributes to the enrichment of bacteria within the primary tumor microenvironment but may also enable some bacteria to enter the circulation alongside exfoliated tumor cells. The resulting bacterial-tumor cell complex may enhance the adaptability of CTCs to the bloodstream environment and alter their interactions with platelets, endothelial cells, and immune cells. This mechanism exhibits strong biological plausibility: on the one hand, abnormal glycosylation of tumor cells provides specific recognition sites for bacterial adhesion; on the other hand, persistent adhesion by *F. nucleatum* can exacerbate local inflammation, promote an invasive tumor cell phenotype, and potentially offer additional protection during the circulatory phase. However, direct evidence regarding whether *F. nucleatum* forms stable complexes with CRC CTCs in the human portal vein remains limited. Therefore, this process should be described in reviews as “a potential mechanism supported by experimental evidence” rather than a fully confirmed clinical event.

Furthermore, metabolic alterations within the tumor microenvironment may further reinforce bacterial-tumor cell adhesion. Infection with *F. nucleatum* can enhance glycolysis and lactic acid accumulation in tumor cells, and lactic acid and its related modifications may influence the expression of tumor cell surface molecules, immune cell function, and the local adhesion environment [24]. These changes may indirectly strengthen the stable interaction between *F. nucleatum* and tumor cells and drive tumor cells toward a more invasive and immune-evasive phenotype.

#### 4.1.2. Formation of the Bacterial-Platelet-Tumor Cell Complex

Once CTCs enter the bloodstream, platelets become one of their most crucial protective companion cells. Platelets rapidly adhere to the surface of tumor cells, forming platelet-encapsulated structures that aid tumor cells in resisting blood flow shear forces, avoiding anoikis, and reducing the likelihood of recognition by NK cells and other immune effector cells [68,69]. In the context of CRC liver metastasis, tumor-associated bacteria may further amplify the platelet-mediated pro-metastatic effects. Lipopolysaccharides, outer membrane proteins, and Fap2 on the surface of *F. nucleatum* can activate platelet-related receptors and procoagulant signals, including TLR4, GPIIb/IIIa, and their downstream calcium signaling and granule release processes [23]. Upon activation, platelets release various adhesion and pro-metastatic factors such as ADP, thromboxane, fibrinogen, fibronectin, TGF-β, and PDGF, promoting platelet aggregation and enhancing their binding to tumor cells. The resulting bacterial-platelet-tumor cell complex structure may provide both physical and immunological protection for tumor cells during circulation. This complex may facilitate the survival of CTCs through at least three mechanisms. (1) Platelet encapsulation buffers the shear forces of portal vein blood flow, reducing tumor cell membrane damage and mechanical apoptosis. (2) Fibronectin, integrin ligands, and procoagulant molecules released by platelets enhance the adhesion between tumor cells and hepatic sinusoidal endothelium and the extracellular matrix, creating favorable conditions for liver retention and colonization. (3) The platelet coating physically impedes direct interactions between natural killer (NK) cells and CD8+ T cells with tumor cells, while concurrently suppressing immune effector functions through the secretion of transforming growth factor-beta (TGF-β) and additional mediators, thereby facilitating immune evasion [24,68,69].

Additionally, platelet-derived signals can directly influence tumor cell phenotypes. TGF-β, PDGF, and related adhesion signals activate pathways such as FAK, PI3K/AKT, and MAPK, enhancing tumor cell migration, anti-apoptotic capabilities, and liver colonization potential [24]. The concurrent activation of the coagulation system and fibrin deposition may further stabilize the bacterial-platelet-tumor cell complex, facilitating the retention of CTCs in the narrow blood flow regions of hepatic sinusoids. However, the bacterial-platelet-tumor cell complex should still be regarded as a mechanistic model requiring further validation. While previous studies have separately supported the adhesion of *F. nucleatum* to CRC cells, the protection of CTCs by platelets, and the metastasis-promoting effects of the coagulation system, whether these three elements form a continuous and stable pro-metastatic structure in the portal vein circulation of human CRLM remains to be confirmed through microfluidic models, portal vein blood sample analysis, CTC capture, and in situ bacterial verification.

#### 4.1.3. Coagulation System and Microemboli Stability

The interaction between CTCs and platelets is frequently accompanied by the activation of the coagulation system. The expression of tissue factors on the surface of tumor cells, the release of platelet granules, and the stimulation by bacteria-related molecules can collectively promote the coagulation cascade, induce fibrin deposition, and form microemboli-like structures [69]. These microemboli not only provide mechanical protection for CTCs but also facilitate their retention within the hepatic microcirculation.

Against the backdrop of hepatic portal vein reflux, the slow blood flow velocity and narrow lumen of the hepatic sinusoids naturally favor the retention of tumor cells, platelets, and fibrin complexes. If *F. nucleatum* or other tumor-associated bacteria are simultaneously present within this structure, the LPS and outer membrane proteins they release may further activate local endothelial cells and Kupffer cells, induce the expression of adhesion molecules, and trigger the release of inflammatory factors, thereby enhancing the interaction between tumor cells and the hepatic microenvironment. Consequently, the coagulation system is not merely a bystander in the process of circulation and metastasis; rather, it may form a complex network with microbial signals, platelet activation, and the hepatic sinusoidal microenvironment to promote tumor cell colonization. Future research should further elucidate whether antiplatelet, anticoagulant, or targeted bacterial signal interventions can reduce the risk of liver metastasis in CRC, as well as assess the safety concerns associated with these strategies, such as bleeding, infection, or dysbiosis.

### 4.2. Escape from Circulating Immune Surveillance

#### 4.2.1. Inhibition of NK Cell Function Mediated by the Fap2–TIGIT Axis

NK cells are crucial effector cells responsible for clearing abnormal cells in the circulatory system and liver innate immunity. They can directly kill CTCs via the perforin-granzyme pathway and enhance anti-tumor immune responses by secreting cytokines such as IFN-γ [70]. During CRC liver metastasis, the ability of CTCs to evade NK cell clearance is a critical prerequisite for their successful entry into the liver and subsequent colonization.

The Fap2 protein of *F. nucleatum* not only participates in tumor cell adhesion but also interacts with the inhibitory receptor TIGIT on the surface of NK cells and certain T cells [70,71]. TIGIT is an important immune checkpoint molecule, and its activation is typically associated with a decline in the effector functions of NK cells and T cells. Upon binding to TIGIT, Fap2 may trigger TIGIT-related inhibitory signals, reducing NK cell activation, degranulation capacity, and the release of perforin and granzyme, thereby weakening their killing effect on CTCs. From a downstream mechanistic perspective, TIGIT activation can affect NK cell activation-related pathways such as PI3K/AKT and MAPK/ERK, and may limit cytotoxic responses through intracellular inhibitory signaling molecules. Concurrently, the Fap2–TIGIT interaction may also diminish the recognition ability mediated by activating receptors such as NKG2D and NKp30 on the surface of NK cells, making it more difficult for tumor cells to be promptly cleared by the immune system [70,71]. This mechanism provides a clear molecular explanation for how *F. nucleatum* promotes immune escape in CRC.

It should be noted that while the role of the Fap2–TIGIT axis in immunosuppression at primary CRC sites has garnered considerable attention, whether it directly operates around CTCs, particularly in weakening NK cell clearance within the portal vein environment, remains to be further confirmed. In the future, the functional contribution of this pathway in the circulating colonization of CRLM can be verified through the detection of NK cell function in portal vein blood, analysis of *F. nucleatum*-positive CTCs, Fap2 blocking experiments, and TIGIT inhibition strategies.

#### 4.2.2. Multiple Inhibition Mediated by the Flora-Metabolism-Immune Axis

Imbalance in the gut microbiota not only affects local intestinal inflammation but also triggers systemic immune regulation by allowing microbial-associated molecules and metabolites to enter the circulation. When the intestinal barrier is compromised, microbial components such as LPS, peptidoglycan, and bacterial DNA can activate myeloid cells, promoting the release of inflammatory factors like TNF-α, IL-6, and IL-1β, and inducing the proliferation of MDSCs, Treg cells, and immunosuppressive macrophages [72]. These systemic immune alterations create an environment in which CTCs can more easily evade immune clearance.

*F. nucleatum* can also contribute to immune evasion through metabolic reprogramming. Studies indicate that *F. nucleatum* enhances glucose uptake and glycolysis in tumor cells, leading to increased lactic acid production [24]. Lactic acid is not only a byproduct of tumor metabolic reprogramming but also a significant immune regulatory factor. It inhibits the proliferation, cytotoxicity, and cytokine secretion of NK cells and CD8+ T cells, promotes the infiltration of MDSCs and Treg cells, and further weakens the anti-tumor immune response through inhibitory molecules such as Arg-1, iNOS, IL-10, and TGF-β [24,72]. Additionally, flora imbalance may affect the antigen-presenting capacity of tumor cells. Some studies suggest that microbial signals can modulate MHC-I molecule expression in tumor cells, reducing their immunogenicity and thus diminishing recognition by NK cells and T cells [73]. During the circulating phase, this reduced antigen presentation may facilitate CTC evasion of immune surveillance; during liver colonization, it may promote the establishment of an immune-privileged state in early micrometastases.

Apart from *F. nucleatum*, other factors, including other flora members, enterovirus co-infections, and drug-resistant bacterial infections, may also indirectly influence CTC survival and liver colonization by altering the inflammatory factor profile, metabolic environment, and immune cell status [74,75]. For instance, enterovirus infections may disrupt flora homeostasis and shift the balance between host antiviral and anti-tumor immunity; drug-resistant bacterial infections may reshape the circulating immune landscape under specific treatment conditions, affecting tumor progression and treatment response. However, the direct causal relationship between these factors and CRLM remains unclear, and excessive extrapolation should be avoided.

#### 4.2.3. Abnormal Function of Local NK Cells in the Liver and the Formation of the Colonization Window

Upon arrival in the liver, CTCs first encounter an immune barrier composed of hepatic sinusoidal endothelial cells, Kupffer cells, NK cells, and NKT cells. Under normal conditions, liver NK cells exhibit robust tumor surveillance capabilities, identifying and eliminating abnormal cells that enter the hepatic sinuses. However, under the combined influence of persistent intestinal microbial signals and tumor-derived factors, local NK cells in the liver may undergo changes such as insufficient recruitment, reduced activation receptors, elevated exhaustion markers, and weakened cytotoxicity [76,77,78].

Abnormalities in chemotactic signals, such as CXCR3, may impair the recruitment and localization of NK cells to the liver, thereby reducing the immune pressure faced by tumor cells upon reaching the hepatic sinuses [77]. Functional remodeling of NK cell subsets within liver metastases may also occur, characterized by diminished anti-tumor effects and enhanced immunosuppressive features [78]. Furthermore, the expansion of MDSCs, Tregs and immunosuppressive macrophages in the liver can further inhibit the function of NK cells and CD8+ T cells through molecules such as IL-10, TGF-β, Arg-1, and iNOS, thereby creating an immune window conducive to the early colonization of tumor cells.

Continuous activation of immune checkpoint molecules also represents a crucial mechanism of immune escape during liver colonization. Molecules such as TIGIT, PD-1, TIM-3, and LAG-3 collectively restrict the function of effector T cells and NK cells, leading to inadequate anti-tumor immune responses [79,80]. With the involvement of microorganisms like *F. nucleatum*, immune checkpoint inhibition may not only originate from tumor cells themselves but also be modulated by bacterial molecules, metabolites, and inflammatory signals. This suggests that the interaction between microorganisms and immune checkpoints may provide an important explanation for the poor response to immunotherapy observed in some patients with CRC liver metastasis.

### 4.3. Exosome-Mediated Amplification of Circulating Signals

#### 4.3.1. Tumor-Derived Exosomes Promote Platelet and Coagulation System Activation

Exosomes serve as crucial carriers for long-distance communication among tumor cells, capable of transporting proteins, lipids, mRNA, microRNA, metabolic enzymes, and inflammation-related molecules to regulate the functions of immune cells, endothelial cells, and platelets in the circulation [68]. During the process of CRC liver metastasis, tumor-derived exosomes can promote platelet activation and initiate the coagulation system, thereby providing a more favorable survival environment for CTCs. Certain exosomes can carry procoagulant molecules, integrin-related proteins, and inflammatory mediators to induce platelet aggregation, fibrin deposition, and the expression of endothelial adhesion molecules [68,69]. These effects enhance the interactions between CTCs and platelets, endothelial cells, and the extracellular matrix, thereby improving their retention capacity in the liver microcirculation. If tumor cells are infected with or chronically colonized by *F. nucleatum*, the contents of the exosomes may undergo further alterations, carrying more signal molecules associated with inflammation, metabolic reprogramming, and immune evasion.

#### 4.3.2. Microbial-Related Exosome Signals and Immune Evasion

Following interaction with tumor cells, *F. nucleatum* may modify the molecular composition of tumor-derived exosomes, enabling them to carry bacteria-related proteins, bacteria-induced microRNA, lactate metabolism-related enzymes, or immunosuppressive signals [68]. Upon entering the circulation, these exosomes can remotely regulate the functions of NK cells, T cells, macrophages and MDSCs, thereby amplifying the systemic effects of local microbial actions. For instance, Fap2-related components carried by exosomes or immune regulatory molecules induced by *F. nucleatum* may participate in inhibiting NK cell activation; metabolic enzymes and lactate-related signals within exosomes may enhance the infiltration of immunosuppressive cells; exosomal microRNA can target genes related to NK cell or T cell activation, promoting effector cell exhaustion or dysfunction [68,72]. These mechanisms collectively constitute an exosome-mediated network for amplifying immune evasion signals. However, exosome-related mechanisms still face numerous methodological challenges. The separation purity, marker definitions, and functional verification standards for exosomes from different sources are not fully standardized; whether bacterial components truly exist within tumor-derived exosomes also requires confirmation through rigorous contamination control and spatial localization methods. Therefore, it should be emphasized in reviews that exosomes are potentially important signal carriers, but their specific cargo composition, target cells, and functional effects still require further validation.

#### 4.3.3. Synergy Among Exosomes, CTCs, and the Liver Microenvironment

Exosomes not only function during the circulatory phase but may also modify the microenvironment of target organs prior to liver colonization. Exosomes released from primary CRC sites can be internalized by Kupffer cells, hepatic sinusoidal endothelial cells, and hepatic stellate cells, triggering the release of inflammatory factors, the expression of adhesion molecules, extracellular matrix remodeling, and the recruitment of immunosuppressive cells [68]. When CTCs subsequently reach the liver, the hepatic environment that has been remodeled by exosomes and intestinal microbial signals may be more conducive to their retention and survival. Therefore, exosomes can be considered a crucial bridge connecting the primary tumor, the circulatory system, and the liver pre-metastatic microenvironment. There may be bidirectional regulation between *F. nucleatum* and other microbial factors: microorganisms can alter the contents of tumor cell exosomes, while exosomes further amplify the inflammatory, coagulatory, and immunosuppressive effects induced by microorganisms. This networked mechanism helps explain why alterations in the local gut microbiota may have systemic metastasis-promoting effects.

### 4.4. Integrated Model and Evidence Boundaries of Microbial Involvement in Liver Colonization

In vitro cellular assays have uncovered several intrinsic molecular axes that govern proliferation, apoptosis, invasion and migratory phenotypes of colorectal-cancer cells. For example, linc01615 acts as an oncogenic long intergenic non-coding RNA exerting reciprocal negative regulation with tumor-suppressive miR-491-5p to shape malignant behaviors in CRC cell culture systems [81]. Of note, these regulatory observations are restricted to cell-culture experiments and have not yet been validated in in vivo CRLM-relevant models.

Similarly, in vitro investigations using colorectal cancer cell lines demonstrate that liraglutide, a GLP-1 receptor agonist, can suppress CRC cell proliferation, migration and invasion while promoting apoptosis, partly via repression of the PI3K/Akt/mTOR signaling cascade [82]. Still, such anti-tumor-related phenotypic effects remain to be interrogated within experimental systems that recapitulate colorectal-cancer-derived liver metastasis.

Based on existing research, microbial involvement in the circulatory colonization process of CRC liver metastasis may encompass the following sequential steps. First, *F. nucleatum* and other tumor-associated bacteria adhere to CRC cells through Fap2–Gal-GalNAc interactions and may enter the portal vein circulation alongside tumor cells. Second, bacterial components can promote platelet activation and initiate the coagulation system, enhancing the anti-shear capacity, immune isolation, and hepatic sinusoidal retention of CTCs. Third, the Fap2–TIGIT axis, lactate metabolism, MDSCs/Treg expansion, and immune checkpoint activation collectively weaken the circulating immune clearance mediated by NK cells and CD8+ T cells. Finally, tumor-derived exosomes and microbial-induced exosome signals can further amplify platelet activation, coagulation, immunosuppression, and pre-metastatic microenvironment remodeling in the liver, thereby increasing the likelihood of CTC colonization in the liver. This model effectively integrates multiple mechanisms, including bacterial adhesion, platelet protection, immune evasion, metabolic inhibition, and exosome communication, but the levels of evidence still need to be clearly distinguished. The interactions between Fap2–Gal-GalNAc and Fap2–TIGIT have a well-defined molecular basis; there is also strong tumor biological evidence supporting platelet protection of CTCs and coagulation-promoted metastasis; and the regulation of metabolism and immune evasion by *F. nucleatum* has some support in CRC. However, whether the bacterial-platelet-tumor cell ternary complex remains stable in the human portal vein circulation, whether *F. nucleatum*-positive CTCs are more prone to forming liver metastases, and whether blocking the aforementioned pathways can reduce the incidence of CRLM remain unresolved issues. Once CTCs overcome circulatory selective pressure and arrive at the liver, they will encounter the pre-remodeled hepatic microenvironment shaped by portal-derived microbial signals, as introduced in Section 3, paving the way for subsequent metastatic-lesion establishment described in Section 5.

Future research should enhance verification in the following areas. (1) Establish a combined detection system for CTCs, platelets, and bacteria in portal vein blood to ascertain whether *F. nucleatum* colocalizes with CTCs. (2) Employ a microfluidic model to simulate the shear stress of portal vein blood flow and observe the formation and stability of bacterial-CTC-platelet complexes. (3) Utilize single-cell sequencing, spatial transcriptomics, FISH and immunofluorescence colocalization techniques to trace the bacterial migration pathways between primary tumors, circulating cells, and liver metastases. (4) In animal models, individually block Fap2–Gal-GalNAc, Fap2–TIGIT interactions, platelet activation, and exosome release to evaluate their functional contributions to liver metastasis formation. (5) Integrate fecal microbiota, circulating microbial DNA, CTC phenotypes, platelet activation markers, and liver metastasis outcomes in clinical cohorts to develop a more predictive metastasis risk model.

In summary, the circulatory phase is not merely a passive transport stage in CRC liver metastasis but a dynamic process involving extensive interactions among tumor cells, microorganisms, platelets, immune cells, the coagulation system, and the exosome network. Tumor-associated bacteria such as *F. nucleatum* may enhance the survival and liver colonization capabilities of CTCs through mechanisms including specific adhesion, platelet microthrombus formation, NK cell inhibition, metabolic immune evasion, and exosome signal amplification. Although these mechanisms offer new insights into the organ-specific metastasis of CRLM, current evidence primarily relies on mechanistic studies and indirect clinical associations, with insufficient dynamic evidence from human studies. Therefore, future research should further elucidate the true role of microorganisms in the circulating colonization stage of CRC liver metastasis through multi-omics, spatial localization, and functional intervention studies, while strictly controlling for contamination and model extrapolation risks.

## 5. Growth and Maintenance of Metastatic Lesions

Following CTC extravasation and early hepatic colonization described in Section 4, established colorectal-cancer-derived liver metastatic lesions exhibit sustained proliferation and heterogeneous therapeutic responses, which are not driven merely by intrinsic neoplastic cell properties but are coordinately shaped by local hepatic immune cells, stromal matrix components, metabolites, and lesion-associated microbial communities specific to CRLM. Advances in tumor microbiomics have documented distinct microbial signatures within paired primary CRC and corresponding hepatic metastatic lesions of colorectal origin. These microorganisms participate specifically in sustaining CRLM lesion progression by rewiring intralesional metabolism, local immune phenotypes, and therapeutic sensitivity. Nevertheless, human CRLM liver metastatic specimens represent typical low-biomass microbial samples, such that microbiome profiling results are highly vulnerable to reagent-borne contamination, sequencing batch artifacts, and tissue-processing biases. Strict methodological controls are therefore mandatory when interpreting observations derived from CRLM metastatic tissues. From the gut–liver axis mechanistic framework established in previous chapters, two major origins shape the intralesional microecology within mature CRLM foci: first, primary-tumor-associated microbes may co-migrate together with disseminated CRC tumor cells and persist within liver metastatic sites; second, gut-derived microbial mediators and metabolites keep flowing into the liver through portal-venous circulation, continuously remodeling the immune-metabolic landscape of already-formed hepatic metastatic lesions. Accordingly, the microecology inside CRLM lesions cannot be regarded as an isolated intra-tumoral event; it arises from continuous crosstalk linking primary CRC, gut commensal communities, and the host hepatic microenvironment.

### 5.1. Evidence of Existence and Functional Significance of the Intratumoral Microbiome

#### 5.1.1. Similarity in Microbial Composition Between Primary Lesions and Liver Metastases

High-throughput sequencing, metagenomics, and multi-omics analyses have provided new insights into the microbial associations between primary CRC and liver metastases. Studies on paired samples have revealed similar microbial compositions in primary CRC and corresponding liver metastases, including *Fusobacterium nucleatum*, *Bacteroides, Prevotella*, and other tumor-associated bacteria (Table 2). This similarity suggests that certain microorganisms may migrate from the primary tumor to distant organs alongside tumor cells and persist in metastatic lesions. This finding holds significant importance. On one hand, it supports the concept of “tumor-associated microbiota migration,” indicating that microorganisms may not merely appear passively in distant tissues but may actively participate in the tumor metastasis process. On the other hand, the similarity in microbial composition between primary tumors and liver metastases suggests that microbial characteristics may reflect the origin, invasive properties, and metastatic pathways of tumor cells. In particular, the co-enrichment of *F. nucleatum* and other bacteria in primary and metastatic CRC provides crucial clues for understanding the involvement of microorganisms in liver metastasis [16,83,84]. However, it should be noted that similarity in microbial composition does not equate to causal evidence. The detection of identical or similar microbial communities in primary and metastatic lesions may result from tumor cell carriage, portal vein input, local contamination, or technical background noise. Therefore, high-resolution spatial localization and functional verification are essential for confirming the true presence and role of microorganisms within tumors.

#### 5.1.2. Potential Functional Roles of Intratumoral Microorganisms

Within CRLM hepatic metastatic foci, lesion-resident microorganisms are not merely passive bystanders. Available evidence suggests that bacteria harbored inside colorectal-cancer liver-metastatic lesions can contribute to local lesion outgrowth and persistence via multiple CRLM-relevant mechanisms. Firstly, these microbes secrete short-chain fatty acids, bile-acid derivatives, lactic acid, lipopolysaccharides and other metabolites that directly or indirectly modulate proliferation, apoptosis, stem-cell-like properties and invasive potential of CRC metastatic tumor cells within the liver microenvironment [85]. Secondly, microbial-derived agonists can trigger TLR, NOD-like receptor, NF-κB, STAT3, and PI3K/AKT signaling cascades, driving inflammatory reprogramming specifically within CRLM metastatic cells. Thirdly, intralesional bacteria shape local immune-cell infiltration and functional states inside hepatic metastatic sites, amplifying the activity of MDSCs, Tregs and immunosuppressive macrophages while dampening CD8+ T-cell, NK-cell and NKT-cell-driven anti-tumor immunity within the liver metastatic niche.

Analysis of some clinical samples has revealed that the abundance of microorganisms or the presence of specific bacterial communities in liver metastases may correlate with the tumor proliferation index, angiogenesis, tumor burden, and poor prognosis [85]. Single-cell sequencing, spatial transcriptomics, and immune co-localization techniques have also started to unveil that microorganisms may be distributed among tumor cells, immune cells, and the stromal region, influencing the metastatic ecosystem through local interactions [86]. These findings suggest that intratumoral microorganisms may contribute to the maintenance of liver metastases through metabolic, immunological, and stromal remodeling processes.

However, most of the current evidence remains correlational, and studies conclusively demonstrating that specific bacterial communities exhibit sustained metabolic activity within liver metastases and directly drive tumor growth are still limited. Therefore, when discussing the functional roles of intratumoral microorganisms, caution should be exercised to avoid equating “microorganism detection” directly with “microorganisms promoting metastasis.” Future research should employ methods such as live bacterial culture, transcriptional activity assays, in situ metabolic imaging, and bacterial community depletion and replenishment experiments to further validate their functions.

#### 5.1.3. Contamination Challenges in Low-Biomass Samples

One of the major methodological challenges in studying intratumoral microorganisms is contamination in low-biomass samples. Unlike intestinal fecal samples, the microbial load in liver tissues and liver metastases is typically low, making sequencing results susceptible to contamination from reagents, environmental bacteria, sample processing procedures, surgical instruments, and batch effects [87,88]. Under such circumstances, conventional 16S rRNA sequencing or metagenomic sequencing may yield false-positive results, leading to an overestimation of the presence and abundance of microorganisms within tumors. Therefore, high-quality research on intratumoral microorganisms should at least adhere to the following methodological standards: implementing strict blank negative controls and reagent controls; correcting for batch effects and filtering out contaminated bacteria; incorporating technical replicates and independent cohort validations; using FISH, immunofluorescence, in situ hybridization, or spatial transcriptomics to confirm the spatial localization of microorganisms within tissues; and, when feasible, conducting live bacterial culture or metabolic activity assays [89,90]. Only through cross-validation using multiple techniques can we reliably determine whether microorganisms in liver metastases are genuinely present and biologically significant.

Thus, the intratumoral microbiome represents a highly promising yet cautiously interpretable avenue in the study of CRC liver metastasis. Future research should transition from merely describing bacterial community composition to conducting comprehensive analyses of in situ localization, functional activity, causal verification, and clinical relevance.

### 5.2. Remodeling of Bile Acid Metabolism and Metastasis Adaptation

#### 5.2.1. Gut Microbiota Regulates Bile Acid Metabolism

Bile acids constitute key gut–liver-axis-derived metabolic mediators that participate in CRLM progression. Their hepatic synthesis, intestinal biotransformation, and enterohepatic circulation depend on coordinated functions of the liver and gut microbial communities. Primary bile acids synthesized in the liver are secreted into the intestinal lumen via biliary excretion. Within the gut, bacterial enzymes including bile-salt-hydrolase (BSH) and 7α-dehydroxylase drive deconjugation and structural modification of primary bile acids, yielding secondary bile-acid species such as deoxycholic acid and lithocholic acid [17]. Changes in gut microbial composition can therefore reshape systemic bile-acid pools and further regulate metabolic homeostasis, inflammatory status, and immune function via nuclear-membrane receptors including FXR, TGR5, and PXR. In the clinical context of CRLM, loss of BSH-expressing commensal bacteria in gut microbiota disturbs this physiological biotransformation process, leading to elevated circulating conjugated primary bile acids (CPBAs) such as taurocholic acid (TCA). These gut-originated bile-acid metabolites travel through portal-venous blood flow and can exert biological effects on both hepatic immune compartments and established liver metastatic foci [16].

During the onset and progression of CRC, flora imbalance may lead to changes in the bile acid metabolic profile. Some studies indicate that patients with CRC or CRC liver metastasis may exhibit elevated levels of conjugated primary bile acids, altered proportions of certain secondary bile acids, and abnormal bile acid receptor signaling [16,17]. These alterations can affect intestinal barrier function, liver immune status, tumor cell metabolism, and inflammatory responses, thereby contributing to the progression of liver metastasis. It is crucial to emphasize that the effects of bile acids on tumors and immunity are significantly influenced by molecular type, concentration, duration of action, and model. Different bile acid molecules may exert distinct or even opposing functions. For instance, certain bile acids may exhibit anti-inflammatory or immunomodulatory effects at low concentrations or in the context of specific receptors, whereas at high concentrations, prolonged exposure, or in inflammatory settings, they may promote DNA damage, oxidative stress, epithelial injury, and tumor progression. Therefore, bile acids cannot be simply categorized as solely pro-metastatic or anti-metastatic factors.

#### 5.2.2. Bile Acid–CXCL16–CXCR6 Axis and Liver Immune Regulation

Changes in bile acid metabolism can influence immune cell recruitment by modulating the expression of hepatic chemokines, with the CXCL16–CXCR6 axis garnering particular attention. CXCL16 is expressed by hepatic sinusoidal endothelial cells, hepatocytes, or tumor-associated cells, while its receptor CXCR6 is highly expressed in liver immune cells such as NKT cells. Variations in bile acid composition can affect CXCL16 expression, thereby regulating the recruitment and function of CXCR6+ NKT cells in the liver [85,88].

In CRLM-relevant experimental models, specific bile-acid species shape NKT-cell-dependent anti-tumor responses within hepatic metastatic microenvironments by modulating CXCL16 expression. Serving as key hepatic immune effectors, NKT-cell subsets exert cytotoxic activity against CRC-derived metastatic tumor cells within the liver through IFN-γ, perforin and granzyme release. Disrupted bile-acid homeostasis may impair NKT-cell recruitment or effector function, dampening liver-intrinsic immune surveillance specifically against CRC metastatic cells and permitting progressive outgrowth of hepatic CRLM lesions [88]. Concurrently, primary or conjugated bile acids may also affect neutrophil infiltration through inflammatory chemotaxis axes such as TIMP1 and CXCL5, indirectly modulating the function of NKT cells and other effector immune cells [16]. This bile acid–chemokine–immune cell network suggests that abnormal bile acid metabolism is not merely a metabolic phenomenon but also serves as a crucial link between flora imbalance and liver immune tolerance.

However, conclusions regarding the relationship between bile acids, CXCL16, and NKT cells vary across studies, potentially due to differences in research models, bile acid types, tumor types, and bile acid composition between animals and humans. Therefore, this axis should be discussed as a significant candidate mechanism in reviews, with the understanding that further validation is required in the human CRLM.

#### 5.2.3. Bile Acid-Mediated Tumor Metabolic Reprogramming

In addition to immune regulation, bile acids can directly influence tumor cell metabolism. They modulate glucose metabolism, lipid metabolism, and mitochondrial function through signaling pathways such as FXR, TGR5, MAPK/ERK, and PI3K/AKT [17,91]. Within the tumor microenvironment, aberrant bile acid metabolism may enhance glycolysis, increase lactic acid production, promote active lipid synthesis, and boost antioxidant stress capacity in tumor cells, thereby aiding metastases in adapting to the liver environment.

Enhanced glycolysis provides energy and biosynthetic precursors for rapidly proliferating tumor cells, while lactic acid accumulation inhibits the functions of CD8+ T cells, NK cells, and NKT cells, and promotes the formation of MDSCs, Treg cells, and immunosuppressive macrophages [91]. The resulting metabolic-immune interactions further reinforce immune tolerance in metastatic lesions. Bile acids may also contribute to the long-term maintenance of metastatic lesions by affecting cholesterol and fatty acid metabolism, altering cell membrane structure, receptor signaling, and tumor cell viability. Therefore, the microbiota-bile acid-tumor metabolic axis may represent a crucial regulatory network for the sustained growth of CRC liver metastases. However, it should be noted that most current studies struggle to distinguish whether alterations in bile acids are the cause, consequence, or merely a concomitant phenomenon of tumor progression. Future research should integrate longitudinal bile acid profiling, microbiota manipulation, receptor-specific blockade, and patient prognosis data to further elucidate these causal relationships.

### 5.3. Microbiome and Treatment Response and Drug Resistance

#### 5.3.1. Intratumoral Microbe-Mediated Drug Metabolism

Intratumoral microorganisms can directly metabolize antitumor drugs, influencing treatment responses—a significant finding in recent tumor microbiome research. Certain bacteria express drug-metabolizing enzymes that convert drugs into low-activity or inactive metabolites, thereby diminishing their antitumor effects [16,92]. For instance, bacteria from the γ-Proteobacteria phylum express cytidine deaminase, mediating the inactivation of gemcitabine; this mechanism has been clearly validated in some tumor models [16]. This suggests that intratumoral microorganisms have the potential to directly alter drug exposure and efficacy.

It should be emphasized that gemcitabine is not a standard core treatment for CRC liver metastases, so its associated evidence serves more as a conceptual basis for understanding how intratumoral microorganisms influence drug metabolism, rather than being directly extrapolated as the primary mechanism of chemotherapy resistance in CRLM. In clinical CRLM treatment, greater attention should be paid to the microbiome’s impact on the responses to 5-fluorouracil, oxaliplatin, irinotecan, anti-EGFR therapy, anti-VEGF therapy, and immunotherapy. Studies have indicated that *F. nucleatum* may be linked to CRC resistance to 5-fluorouracil and oxaliplatin, potentially involving mechanisms such as autophagy activation, enhanced inflammatory signaling, apoptosis inhibition, and the formation of an immunosuppressive microenvironment. Gut microbiota can also influence irinotecan-related enterotoxicity; for example, bacterial β-glucuronidase participates in the reactivation of irinotecan metabolites, exacerbating intestinal damage. These findings suggest that the microbiome not only affects the antitumor efficacy of drugs but also modulates drug toxicity and patient tolerance.

#### 5.3.2. Flora-Driven Multidrug Resistance Phenotype

In addition to directly metabolizing drugs, the flora can also induce multidrug resistance by modulating tumor cell signaling pathways and the microenvironment. Within CRLM hepatic metastatic niches, microbially derived agonists and metabolites trigger NF-κB, STAT3, MAPK, PI3K/AKT and Wnt/β-catenin signaling cascades in CRC metastatic tumor cells, enhancing anti-apoptotic programs, DNA-damage-repair capacity and drug-efflux machinery of liver-colonized colorectal-cancer cells [25,26]. For example, up-regulation of drug-efflux transporters including MDR1, ABCG2 and ABCB1 can lower intracellular drug concentrations inside CRLM metastatic cells and blunt chemotherapeutic efficacy against these liver-residing CRC tumor cells.

Microbial metabolites may also influence DNA damage repair pathways. Certain flora-related signals can induce the expression of repair molecules such as MGMT, BRCA1, and RAD51, enabling tumor cells to more readily repair DNA damage induced by chemotherapy or radiotherapy [26]. Concurrently, flora imbalance can promote the maintenance of tumor stemness and epithelial–mesenchymal transition, endowing tumor cells with enhanced anti-apoptotic, invasive, and treatment-resistant properties [93].

The immune microenvironment also plays a crucial role in microbial-mediated drug resistance. Factors such as *F. nucleatum*, LPS, bile acids, and lactic acid can facilitate the infiltration of MDSCs, Treg cells, and immunosuppressive macrophages, impairing the function of CD8+ T cells and NK cells, and thereby weakening the anti-tumor immune effects induced by chemotherapy, targeted therapy, and immunotherapy. For MSI-H/dMMR CRC patients, the composition of the gut microbiota may influence the efficacy of immune checkpoint inhibitors; for MSS-type CRC, the role of flora in regulating the immune-cold microenvironment may also impact the development of combined immunotherapy strategies.

#### 5.3.3. Microbial-Tumor Interactions Maintain Drug Resistance Homeostasis

Drug resistance is not merely the result of transient activation of a single pathway but rather a steady state formed through long-term interactions among microorganisms, tumor cells, immune cells, and the metabolic network. Gut microbiota and its metabolites can be continuously transported to the liver via the gut–liver axis, sustaining local inflammation, immunosuppression, and metabolic reprogramming [17,91]. Intratumoral microorganisms can disseminate drug resistance-related information among tumor cells through exosomes, metabolites, and inflammatory signals, promoting the spread of drug-resistant phenotypes [94]. Additionally, microbial-related signals may participate in epigenetic regulation, including DNA methylation, histone modification, and alterations in non-coding RNA expression [95]. These changes can stabilize tumor cell stemness, anti-apoptotic properties, and drug efflux capabilities, rendering the drug-resistant state somewhat memorable and difficult to reverse. Flora-immune interactions may also drive tumor evolution under therapeutic pressure by eliminating drug-sensitive clones and retaining drug-resistant ones.

Taken together, the intralesional microecology specific to CRLM shapes therapeutic responses of colorectal-cancer hepatic metastases through interconnected mechanisms covering microbial drug metabolism, oncogenic-signaling-pathway activation, local immunosuppression, metabolic reprogramming and epigenetic modification. Consistent with earlier observations, partially overlapping microbial profiles can be detected between paired primary CRC and matched CRLM hepatic metastatic specimens. Intralesional microbes, bile-acid metabolic rewiring, plus gut-derived signals continuously delivered through the portal-venous gut–liver axis jointly support lesion outgrowth, local immune-tolerant states, and stable drug-resistant phenotypes within CRLM foci. Nevertheless, substantial evidentiary gaps remain in this research area. First, low-biomass features of human CRLM metastatic tissues bring high contamination risk for microbiome profiling. Second, numerous mechanistic findings originate from pre-clinical animal-model systems, in vitro assays, or pan-cancer datasets; direct experimental evidence specifically validated in CRLM patient samples is still sparse. It also remains incompletely disentangled whether microbial alterations represent causal drivers, secondary consequences, or merely epiphenomena accompanying CRLM lesion formation and drug-resistance evolution. Future work should clarify the genuine functional contribution of CRLM-associated microecology to therapeutic outcomes, relying on rigorous contamination-mitigation protocols, spatial in situ localization assays, multi-omics integration, and prospective clinical patient cohorts.

## 6. Clinical Translation and Future Prospects

The gut–liver axis-mediated microbial-immune-metabolic interactions offer novel insights into risk prediction, early monitoring, and precise intervention for CRC liver metastasis. (Figure 1) Compared with traditional tumor markers and imaging assessments, indicators such as gut microbiota, circulating microbial DNA, bile acid profiles, short-chain fatty acids, and microbial-derived exosomes demonstrate potential for non-invasive, dynamic, and repeatable detection. Meanwhile, targeted microecological intervention strategies, including precision antibiotics, phage therapy, postbiotic supplementation, dietary modulation, and combined immunotherapy, also hold promise for blocking the metastatic process or enhancing treatment responses. However, research on microecological translation remains in its infancy. The core challenges currently include significant population heterogeneity, inconsistent detection standards, low biomass contamination, unclear causality, undefined intervention windows, and insufficient long-term safety data. Therefore, the clinical translation section should emphasize potential while thoroughly discussing risk boundaries and evidence gaps to avoid premature clinical application of strategies still in the experimental phase.

### 6.1. Microbiological Markers and Risk Stratification for Liver Metastasis

#### 6.1.1. Fecal Microbiota Markers

Fecal microbiota detection represents one of the most practical microecological assessment methods available. CRC patients often exhibit reduced bacterial diversity, decreased abundance of short-chain fatty acid-producing bacteria, and enrichment of potentially pathogenic or pro-inflammatory bacteria such as *F. nucleatum*, *Bacteroides* and *Prevotella* [96]. These alterations are not only associated with CRC development but may also correlate with liver metastasis risk, recurrence, and prognosis.

*F. nucleatum* stands out as a highly representative candidate marker. Elevated abundance in CRC tissues and feces is linked to tumor aggressiveness, immunosuppression, and poor prognosis [96,97,98]. Combining fecal *F. nucleatum* abundance with serum bile acid profiles, inflammatory markers, CEA, CA19-9, or imaging features may enhance identification of high-risk populations for CRLM. Compared to single markers, multidimensional combined models better align with the complex pathophysiology of CRLM. However, fecal microbiota composition is significantly influenced by diet, antibiotics, geographic region, age, underlying diseases, sampling methods, and sequencing platforms, limiting the stability of individual bacterial species across different cohorts. Thus, future efforts may focus less on identifying a single highly specific bacterial species and more on developing comprehensive risk models integrating microbiota structure, functional genes, metabolites, and clinical variables.

#### 6.1.2. Blood Microbial DNA and Metabolite Markers

Circulating microbial DNA, LPS, bacterial outer membrane vesicles, and microbiota-derived metabolites can reflect the extent of intestinal barrier disruption and microbial translocation, thereby serving as potential indicators for evaluating abnormal activation of the gut–liver axis [97]. Compared with fecal samples, blood markers are more closely associated with systemic inflammation and liver exposure, theoretically providing a more direct reflection of liver metastasis risk.

The DNA profile of circulating microorganisms can be incorporated into liquid biopsies to monitor the risk of postoperative recurrence and distant metastasis [97]. Bile acid profiles, short-chain fatty acids, lactic acid, and other microbial metabolites can also reveal functional alterations in the microbiota at the metabolic level. For instance, elevated levels of conjugated bile acids, abnormal secondary bile acids, and decreased short-chain fatty acids may indicate dysfunctional gut microbiota metabolism and aberrant liver immune regulation [98,99]. Additionally, microbial-related RNA, proteins, or metabolic signals carried by circulating exosomes may serve as supplementary indicators [100]. Exosomes exhibit good stability and can reflect the communication between tumor cells and the microenvironment, thus holding potential value in the early monitoring of CRLM. However, the detection of microbial DNA and exosomes in blood also faces challenges such as low biomass contamination, background noise, lack of standardization, and difficulties in causal interpretation. Large-sample prospective validation is still required before their clinical routine application.

#### 6.1.3. Multi-Index Joint Risk Model

Given that the occurrence of CRLM involves multiple aspects, including tumor cells, host immunity, intestinal barrier, microbiota metabolism, and liver microenvironment, a single microbial marker often falls short of meeting clinical risk stratification needs. A more feasible strategy in the future is to establish a multi-index joint model that integrates fecal microbiota, circulating microbial DNA, bile acid profiles, short-chain fatty acids, inflammatory factors, CTCs, ctDNA, tumor staging, molecular typing, and imaging features for comprehensive analysis.

This type of model is expected to be applied in three clinical scenarios. First, it can identify high-risk populations for liver metastasis in patients with early-stage CRC after surgery, optimizing follow-up frequency and imaging monitoring. Second, it can evaluate metastatic burden, microecological status, and treatment response in patients with synchronous liver metastasis. Third, it can dynamically monitor recurrence risk and microecological recovery after systemic therapy or hepatectomy. However, before practical implementation, issues such as model stability, cross-center reproducibility, detection costs, and clinical interpretability must be addressed. High-quality reviews should emphasize that the value of microbial markers lies not only in their statistical correlations but also in their ability to improve existing clinical decision-making.

### 6.2. Intervention Strategies Targeting Intestinal Microecology

From the clinical-translational perspective, microbiota-targeted interventions for colorectal-cancer-related liver metastasis fall into two distinct application scenarios. The first is prophylactic intervention, which targets high-risk early-stage CRC patients after curative resection without established liver metastasis. Its core goal is to remodel gut microbial composition and metabolic output, sustain intestinal-barrier integrity, and suppress pathological portal-venous microbial translocation so as to lower subsequent CRLM occurrence risk. The second corresponds to therapeutic intervention for patients already diagnosed with colorectal-cancer liver metastasis. Such interventions aim to reshape the gut–liver microecological milieu, improve responsiveness to chemotherapy, targeted therapy or immunotherapy, reduce treatment-related toxic effects, and constrain the progression of metastatic lesions. It should be noted that most microbiota-modulating approaches are still confined to pre-clinical or early exploratory research, and few have been validated in dedicated CRLM patient cohorts. Each intervention modality described below will be assessed from four dimensions: mechanistic rationale, available evidence level (pre-clinical animal models/human observational cohorts/clinical trials), potential advantages, and major translational limitations (Table 3).

#### 6.2.1. Precise Antibiotic Intervention

Antibiotics represent one of the most direct approaches to microecological intervention. Narrow-spectrum, short-course, and individualized antibiotic strategies targeting *F. nucleatum* and other metastasis-promoting bacteria may theoretically reduce pathogen load, alleviate inflammatory responses, improve the immune microenvironment, and enhance treatment sensitivity. For instance, metronidazole and other drugs can decrease the load of anaerobic bacteria and have demonstrated potential in inhibiting the progression of *F. nucleatum*-related tumors in certain studies. Non-absorbable antibiotics may also exert effects by locally regulating the gut microbiota, influencing bile acid metabolism, and reducing intestinal-derived inflammatory inputs. However, antibiotic intervention is a double-edged sword. The use of broad-spectrum or long-term antibiotics may lead to a reduction in commensal bacteria, a decline in short-chain fatty acids, impaired barrier function, the proliferation of drug-resistant bacteria, and a diminished immune response [101,102]. In the context of cancer treatment, inappropriate antibiotic use may even weaken anti-tumor immunity, increase the risk of infection, and induce long-term dysbiosis. Therefore, future antibiotic strategies should emphasize precision rather than extensive bacterial eradication.

More reasonable approaches include: screening target populations based on microbiota profiling; selecting narrow-spectrum or bacteria-promoting-metastasis-targeted drugs; limiting short-term dosing windows; combining with postbiotics or prebiotics to restore barrier function; dynamically monitoring microbiota recovery and drug resistance risks. Only after identifying the beneficiary populations and establishing safety boundaries can antibiotic intervention serve as an adjunctive strategy for the prevention and treatment of CRLM. For prophylactic scenarios in high-risk non-metastatic CRC, narrow-spectrum short-course antibiotics theoretically could deplete metastasis-promoting pathobionts before portal microbial translocation occurs; nevertheless, risks of commensal flora disruption complicate its routine prophylactic application. For therapeutic use in patients with established CRLM, antibiotics may reduce tumor-associated pathogen burden and alleviate local inflammation, yet broad-range perturbation of gut commensals raises safety concerns and lacks dedicated clinical trial evidence in CRLM cohorts.

#### 6.2.2. Phage Therapy

Phage therapy exhibits high specificity, capable of specifically lysing target bacteria while relatively preserving other commensals, making it a more precise microecological intervention than broad-spectrum antibiotics. Specific or engineered phages targeting *F. nucleatum* can theoretically reduce the load of tumor-associated bacteria, block bacterial-tumor cell interactions, alleviate immunosuppression, and enhance responses to chemotherapy or immunotherapy [103].

Phage therapy offers advantages such as strong targeting, high design flexibility, and the potential for combination with nanodelivery systems, antitumor drugs, or immunomodulators. For example, utilizing phages to recognize *F. nucleatum* and deliver antimicrobial molecules or antitumor drugs holds promise for achieving dual targeting of lesion microecology and tumor therapy. However, its clinical translation faces multiple challenges. First, significant heterogeneity exists among different strains of *F. nucleatum*, making it difficult for a single phage to cover all relevant strains. Second, phages can induce host immune responses, generating neutralizing antibodies or inflammatory reactions that affect efficacy and safety. Third, the production quality control, administration routes, dosages, treatment durations, and regulatory standards for phage preparations are not yet fully established [103]. Fourth, whether excessive elimination of a specific bacterial population disrupts the overall microbial ecology requires long-term follow-up evaluation. Therefore, phage therapy is more appropriately viewed as a promising precise intervention approach rather than a mature clinical treatment strategy. In prophylactic settings for high-risk CRC without liver metastasis, target-specific phages could selectively eliminate pathobionts such as *F. nucleatum* while preserving commensal microbes, theoretically lowering the risk of pathological microbial translocation. In therapeutic settings for CRLM patients, phage agents might alleviate bacteria-driven immunosuppression within primary or metastatic lesions; however, strain heterogeneity, anti-phage immune responses, and lack of human CRLM-focused trials constitute major practical bottlenecks.

#### 6.2.3. Probiotics

Probiotics are live microorganisms that confer host health benefits at adequate doses, with most oncological-relevant strains from *Lactobacillus* and *Bifidobacterium* [27]. They competitively suppress pathobionts, reinforce intestinal barrier integrity, mitigate inflammation and boost anti-tumor immunity including NK-cell and CD8+ T-cell responses, while also alleviating cancer-therapy-related mucosal injury [27]. Most supporting data originate from pre-clinical colorectal-cancer animal models; limited human observational and early-phase CRC trials show improvements in gut dysbiosis and chemotherapy-induced gastrointestinal toxicity, yet no dedicated pre-clinical or clinical datasets exist for CRLM.

As multi-modal live-biotic interventions, probiotics modulate whole-gut ecology with relatively low intrinsic toxicity. Still, their translation is hampered by strong strain-specific effects, large inter-individual microbiota-dependent response heterogeneity, lack of standardized oncological dosing protocols, and theoretical bacteraemia risks for immunocompromised patients [27]. Conceptually, probiotics may stabilize gut homeostasis and limit pathological portal-venous microbial translocation for prophylactic intervention in high-risk non-metastatic CRC, but variable responses preclude routine clinical recommendation. For therapeutic intervention in patients with established CRLM, probiotics cannot regress hepatic metastases and are only hypothetically useful as adjuvant support to ameliorate treatment-related gut injury, remaining purely exploratory without CRLM-specific validation.

#### 6.2.4. Prebiotics

Prebiotics are indigestible dietary substrates selectively fermented by commensal bacteria to enrich beneficial taxa such as *Bifidobacterium* and *Lactobacillus*; typical representatives include inulin, fructooligosaccharides and resistant starch [27]. Fermentation-derived short-chain fatty acids strengthen intestinal barrier function, modulate regulatory T-cell activity and suppress oncogenic inflammatory cascades. Available evidence mainly comes from pre-clinical colorectal-cancer animal models, with limited human observational links between prebiotic-rich diet and improved gut homeostasis in CRC patients. No dedicated pre-clinical or clinical evidence is available for CRLM cohorts.

Compared with live-microbe therapies, prebiotics avoid infective safety risks while reshaping gut metabolic profiles. Major translational obstacles include strong response heterogeneity determined by individual baseline microbiota and the absence of unified oncological dosing standards, alongside scarce large-scale cancer-focused trials [27]. Conceptually, prebiotics may build favorable microbiota-metabolite profiles to restrict prometastatic portal-venous mediators for prophylactic intervention in high-risk non-metastatic CRC, yet inter-subject variability prevents routine application. For therapeutic intervention in patients with established CRLM, prebiotics are theoretically feasible as gut-directed adjuvant support for anti-tumor regimens but cannot be prioritized given the complete lack of CRLM-specific validation.

#### 6.2.5. Synbiotics

Synbiotics are rationally designed composite preparations combining probiotics and fermentable prebiotics to generate synergistic biological effects superior to single-component treatment [27]. They stabilize gut microbial diversity, elevate short-chain-fatty-acid production and suppress chronic inflammatory signals, ameliorating dysbiosis-driven pathological changes in pre-clinical colorectal-cancer models. Up to now, no direct human clinical evidence from CRLM cohorts has been reported.

By matching probiotic strains with their preferred nutrient substrates, synbiotics theoretically improve gastrointestinal survival of beneficial microbes and amplify downstream metabolic-immunomodulatory outputs. Nevertheless, translational difficulties remain: formulation requires careful strain-substrate matching, patient-specific microbiota causes heterogeneous responses, and large-scale oncology-focused clinical trials remain scarce [27]. Conceptually, synbiotics may maintain balanced gut ecology and reduce portal-venous microbial-translocation risk for prophylactic intervention in high-risk non-metastatic CRC, yet heterogeneous responses rule out routine prophylaxis. For therapeutic intervention in patients with established CRLM, synbiotics hold theoretical adjuvant value for mitigating treatment-related gut dysbiosis but remain purely exploratory without CRLM-specific supporting data.

#### 6.2.6. Engineered Bacteria

Engineered bacteria refer to genetically modified commensal or attenuated microbial strains equipped with programmable gene circuits, which can produce anti-tumor effectors, therapeutic enzymes or immunomodulatory molecules in situ within the intestinal or tumor microenvironment [28]. Mechanistically, rational genetic modification endows bacterial strains with improved gastrointestinal tolerance, tumor-targeted colonization capacity, and controllable payload-release functions. Apart from directly generating anti-tumor mediators, engineered bacteria can remodel local gut community composition, restore intestinal barrier homeostasis, and boost systemic anti-tumor immune responses [28]. Pre-clinical colorectal-cancer animal-model data demonstrate that well-designed engineered bacterial constructs can suppress tumor proliferation, ameliorate gut dysbiosis, and synergize with chemotherapy or immune-checkpoint-blockade therapy. Nevertheless, dedicated pre-clinical studies and human clinical cohorts focusing specifically on CRLM remain absent.

Compared with conventional wild-type probiotics, genetically engineered bacteria achieve programmable, target-oriented therapeutic output beyond natural microbial functions. Still, multiple substantial translational obstacles persist. Complex genetic circuit design and strain biosafety assessment impose high development thresholds; oral delivery faces harsh gastrointestinal environmental stress that impairs bacterial viability. Moreover, potential risks of uncontrolled in vivo gene transfer, off-target colonization, and opportunistic infection in immunocompromised hosts cannot be ignored, while unified manufacturing and quality-control standards for oncology-oriented engineered bacteria have not yet been fully established [28]. Conceptually, for prophylactic intervention in high-risk non-metastatic CRC, engineered bacterial systems may stabilize gut ecology and restrain pathological portal-venous microbial translocation before circulating tumor-cell hepatic seeding, yet biosafety uncertainties preclude routine prophylactic application. For therapeutic intervention in patients with established CRLM, engineered bacteria hold theoretical adjuvant potential to reshape gut-derived anti-tumor immunity and improve responses to systemic anti-cancer regimens. Owing to the complete lack of CRLM-specific pre-clinical and clinical validation, such interventions remain purely exploratory.

#### 6.2.7. Microbial-Metabolite-Oriented Intervention

Postbiotic and short-chain fatty acid supplementation represent another crucial microecological intervention strategy. Short-chain fatty acids, particularly butyric acid, propionic acid, and acetic acid, are key metabolites produced through the fermentation of dietary fiber by intestinal symbiotic bacteria. These compounds provide energy to intestinal epithelial cells, enhance the expression of tight junction proteins, suppress inflammatory responses, and modulate the functions of Treg cells, macrophages, and CD8+ T cells [104]. Consequently, restoring short-chain fatty acid levels may aid in repairing the intestinal barrier, reducing microbial translocation, and mitigating abnormal activation of the gut–liver axis.

Dietary fiber, inulin, resistant starch, traditional Chinese medicine polysaccharides, and postbiotic preparations may exert their effects by promoting short-chain fatty acid production, improving microbial community structure, and enhancing barrier function [89,104,105,106,107]. These strategies are generally gentler than antibiotics and may offer superior long-term safety, making them promising for postoperative recovery, adjuvant therapy, and management of high-risk populations. However, the effects of short-chain fatty acids are also dose-, time-, and disease stage-dependent. While an appropriate amount of short-chain fatty acids typically helps maintain barrier homeostasis and suppress inflammation, high concentrations or inappropriate timing of supplementation may yield complex or even opposing effects in certain tumor metabolic contexts [104]. Furthermore, variations in the baseline microbial community structure among patients lead to significant differences in their responses to dietary fiber or postbiotics. Therefore, postbiotic interventions should not be universally regarded as beneficial but should be tailored based on the patient’s microbial baseline, treatment stage, and metabolic status.

#### 6.2.8. Bile-Acid-Targeted Therapeutic Strategies

Bile-acid-targeted therapy constitutes another distinct microbial-metabolite-relevant intervention for CRLM, built upon gut–liver-axis pathological mechanisms described in Section 2 and Section 5. Clinical cohort observations and animal-model experiments from Ref. [16] demonstrate that loss of gut bacterial bile-salt-hydrolase (BSH) function drives excessive accumulation of circulating conjugated primary bile acids such as taurocholic acid (TCA), which promotes hepatic metastasis via neutrophil-recruitment-dependent prometastatic signaling [16]. Pre-clinical animal-model evidence within the same study has demonstrated that correction of elevated conjugated primary bile acids can suppress neutrophil-mediated prometastatic cascades by restoring the balance between conjugated and unconjugated bile-acid pools [16].

Conceptually, therapeutic strategies aim to reverse pathological bile-acid pool disturbance, either by restoring the abundance of BSH-producing commensal bacteria or via pharmacological modulation of bile-acid-sensing nuclear receptors. For prophylactic intervention in high-risk non-metastatic CRC, restoring physiological bile-acid profiles may restrain portal-venous delivery of prometastatic bile-acid mediators prior to CTC hepatic seeding. For therapeutic intervention in patients with established CRLM, bile-acid-targeted approaches hold theoretical potential to remodel intra-metastatic immune microenvironments. Nevertheless, direct clinical evidence specifically obtained from human CRLM patient cohorts remains absent in existing literature. Major translational limitations include large inter-individual variability of bile-acid metabolism, context-dependent dual biological effects exerted by different bile-acid species, and technical obstacles to achieving tissue-specific bile-acid modulation within the liver.

#### 6.2.9. Microbiota-Directed Nanomedicine

Microbiota-directed nanomedicine employs nano-scale delivery platforms to achieve precise gut–microbiota modulation, including selective depletion of pro-tumoral pathobionts and targeted delivery of probiotic-related payloads, while overcoming gastrointestinal physiological barriers that limit conventional microbial interventions [108]. Nanocarriers can protect active cargoes from gastric acid and intestinal enzyme degradation, enable targeted elimination of tumor-associated bacteria such as *Fusobacterium nucleatum*, or facilitate colon-specific colonization of beneficial microbes to restore balanced gut ecology. In pre-clinical CRC animal models, such nanosystems have been shown to remodel gut and intratumoral microbiome profiles, relieve chemotherapy resistance, and improve anti-tumor immune responses [108]. However, there are no dedicated pre-clinical or clinical datasets evaluating these nanomedicines specifically within CRLM settings.

Compared with free-drug or conventional microbial supplementation strategies, nanomedicine improves payload stability, target selectivity and local bioavailability, and can simultaneously combine microbiota-modulating agents with chemotherapeutic or immunotherapeutic moieties for synergistic effects. Even so, multiple translational bottlenecks persist. Complex gastrointestinal barriers impose high requirements for material design; intratumoral microbiota targeting remains technically challenging, and individual-specific gut microbial features demand personalized formulation optimization. At present, relevant investigations are confined almost entirely to pre-clinical animal experiments, with no mature human clinical trial data available [108]. Conceptually, for prophylactic intervention in high-risk non-metastatic CRC, microbiota-directed nanomedicine may selectively clear prometastatic pathobionts and stabilize gut microbial homeostasis to reduce the risk of portal-venous microbial translocation, yet insufficient clinical validation prevents routine preventive adoption. For therapeutic intervention in patients with established CRLM, these nanoplatforms theoretically offer potential as combinatory adjuvant approaches to ameliorate microbiota-driven chemoresistance and reshape gut–liver immune crosstalk. Nevertheless, without CRLM-oriented pre-clinical and clinical validation, such applications remain purely exploratory.

#### 6.2.10. Combined Intervention Strategies

Single microecological interventions often fall short of completely halting the complex progression of CRLM. A more promising approach in the future may involve combined interventions, such as using precision antibiotics to eliminate metastasis-promoting bacteria followed by postbiotics or dietary fiber to restore symbiotic bacterial ecology; combining phage therapy with chemotherapy and immunotherapy to enhance anti-tumor effects [100,109].

The advantage of combined strategies lies in their ability to simultaneously target bacterial load, barrier function, immune status, and tumor cell resistance. However, the associated risks are more complex, encompassing drug interactions, excessive microbial community disturbance, immune activation, toxicity of the delivery system, and uncertain long-term safety. Therefore, before clinical application, it is essential to determine the optimal combination, dosage, timing, and applicable population through animal models, organoid-microbiota co-culture systems, and early-phase clinical trials.

When considering prophylactic application for high-risk non-metastatic CRC, combined regimens should prioritize safety and avoid excessive perturbation of commensal gut flora. Under therapeutic conditions for patients with established CRLM, combined microecological approaches need to be carefully balanced against anti-tumor regimens, so as not to amplify adverse events. At present, there remains a lack of dedicated clinical trial data supporting combined microbiota-targeted regimens for either scenario.

**Table 3:** Overview of gut–microbiota-targeted intervention modalities for colorectal-cancer-derived liver metastasis.

Therapeutic Intervention	Core Mechanistic Rationale	Clinical Readiness	Main Risks and Limitations	Level of Supporting Evidence	References
Probiotics	Restore intestinal-barrier integrity; suppress expansion of pathogens and reduce portal-venous pathological microbial translocation.	Exploratory adjuvant concept; partial human safety data from general gastrointestinal disease cohorts; no large-scale dedicated RCTs for CRLM high-risk postoperative patients.	Therapeutic efficacy is strongly strain-dependent; bacteraemia risk for immunocompromised patients; mainly acts within intestinal lumen and cannot eliminate intralesional pathogens inside established hepatic metastatic nodules.	Pre-clinical in vitro/murine mechanistic data + partial human observational safety data (non-CRLM cohorts); no CRLM-specific efficacy clinical evidence.	[27]
Prebiotics	Promote growth of beneficial commensal microbes, boost protective microbial-metabolite production, improve gut-barrier function and suppress overgrowth of pathogens.	Exploratory adjuvant concept; partial human safety background from gastrointestinal-disease cohorts; no large-scale CRLM-focused clinical trials.	Inter-patient response variability driven by individual baseline gut–microbiota composition.	Pre-clinical mechanistic data + partial human observational safety data (non-CRLM cohorts); no definitive CRLM-specific efficacy evidence.	[27]
Engineered bacteria	Genetically modified bacterial platforms for targeted delivery of anti-tumor payloads inside tumor microenvironments.	Strictly pre-clinical-stage; zero human clinical data specific for CRLM.	Biosafety risks including horizontal gene transfer, off-target bacterial translocation; poor penetration into well-established hepatic metastatic lesions.	Only murine pre-clinical proof-of-concept results; no human-derived CRLM clinical evidence.	[28]
Microbial metabolite modulation	Supplement functional microbial-derived metabolites (short-chain-fatty-acid-based postbiotic interventions); repair intestinal barrier, suppress gut-derived pro-inflammatory signaling and remodel hepatic immune microenvironment.	Exploratory adjuvant direction; partial human safety data from gastrointestinal cohorts; no large-scale prospective clinical validation in CRLM.	Biological effects are dose-, timing-, and disease-stage-dependent; substantial inter-patient variability in metabolic responses.	Pre-clinical mechanistic data + partial human observational safety data (non-CRLM cohorts); lack definitive CRLM-specific efficacy evidence.	[89,104,105,106,107]
Bile acid-targeting therapies (FXR/TGR5 modulators)	Modulate bile acid-receptor signaling pathways to remodel MDSC-dominated immunosuppressive hepatic microenvironment.	Pre-clinical stage only; insufficient CRLM-patient-specific c clinical-cohort evidence.	Drug effects are context-, dose- and tissue-dependent; potential tumor-promoting risk; findings obtained from cholestatic liver-disease models cannot be directly extrapolated to CRLM patients.	Mainly pre-clinical murine/organoid-model mechanistic results; no robust human CRLM clinical efficacy evidence.	[16,17,91]
Microbiome-directed nanomedicine	Nanocarrier-mediated targeted delivery of phages, antimicrobials, bacterial metabolites, or immunomodulators, aiming to reduce systemic off-target adverse effects.	Exclusively pre-clinical research stage; no prospective human clinical data for CRLM patients.	Risk of off-target hepatic inflammation; material immunogenicity; challenges for large-scale manufacturing standardization; unsatisfactory in vivo biodistribution of general oncology nanocarriers.	Only pre-clinical animal-model findings; no human clinical evidence for CRLM.	[108]

Notes: CRLM, colorectal-cancer liver metastasis; RCTs, randomized controlled trials; FXR, farnesoid X receptor; TGR5, Takeda G protein-coupled receptor 5.

### 6.3. Current Research Limitations

Although research on the gut–liver axis microecology offers a fresh perspective for the prevention and treatment of CRLM, several critical limitations persist.

Firstly, there is significant population heterogeneity. The composition of the gut microbiota is profoundly influenced by factors such as geographical location, diet, age, medication use, tumor site, treatment modalities, and underlying diseases, resulting in limited reproducibility of findings across different cohorts [96]. Secondly, detection techniques lack standardization. Variations in fecal sample collection, DNA extraction methods, sequencing platforms, bioinformatics analyses, and contamination control procedures can lead to discrepancies in results. This issue is particularly pronounced for low-biomass samples, including blood, liver tissue, and intratumoral microorganisms, where contamination poses a significant challenge [96,97]. Thirdly, the causal relationships remain ambiguous. Most clinical studies employ cross-sectional or retrospective correlation analyses, making it difficult to ascertain whether alterations in the gut microbiota are the cause, consequence, or merely a coincidental phenomenon associated with liver metastasis. Fourthly, obtaining pre-metastatic human samples is challenging. Hepatic pre-metastatic niches are crucial for understanding the early events of CRLM; however, direct sampling and verification in humans are difficult, leading to a heavy reliance on animal models for current mechanistic evidence. Fifthly, the safety margins of intervention strategies are unclear. Antibiotics, bacteriophages, short-chain fatty acids, and combined interventions may exert bidirectional effects, and their long-term safety and appropriate patient populations require further clarification [101,102,103]. Lastly, there is a dearth of evidence for clinical translation. Most studies are currently confined to mechanistic explorations or small-scale validations, lacking multicenter, large-scale, prospective clinical trials to demonstrate that microecological markers or intervention strategies can genuinely improve outcomes for CRLM patients.

### 6.4. Future Research Directions

In the future, research on the gut–liver axis in CRLM should transition from descriptive correlation to mechanism verification and clinical application, with a focus on the following directions.

(1) Spatial multiomics research should be intensified. Spatial transcriptomics, spatial proteomics, spatial metabolomics, and spatial microbiomics can elucidate the spatial relationships among microorganisms, tumor cells, immune cells, and stromal cells at the tissue in situ level, thereby determining whether microorganisms are truly situated within the tumor niche and functionally interacting with specific cell populations [109,110]. (2) A longitudinal clinical cohort should be established. By dynamically collecting stool, blood, tumor tissue, and clinical outcome data throughout the processes of CRC diagnosis, surgery, adjuvant therapy, recurrence, and liver metastasis, we can more precisely evaluate the temporal relationship between microbiota changes and liver metastasis [99]. (3) Multiomics and artificial intelligence modeling should be integrated. A combined analysis of metagenomic, metabolomic, transcriptomic, immune, ctDNA, CTC, and radiomic data holds promise for establishing a more stable CRLM risk prediction model and identifying key microbiota and metabolic pathways with genuine intervention potential [111]. Multi-compartment multi-omics strategies, which simultaneously profile gut luminal contents, circulating biofluids and distal target-tissue metabolome-microbiome signatures, have been implemented in pre-clinical disease models to map cross-organ microbiota-metabolite communication networks [112]. Notably, this cited methodological demonstration was performed in an Alzheimer’s-disease mouse model rather than CRLM-relevant experimental systems; nevertheless, such multi-sample-source analytical frameworks deliver valuable methodological reference for future gut–liver-axis mechanistic investigations in CRLM. (4) Experimental models that more closely resemble the human body should be developed. Models such as organoid-microbiota co-cultures, liver-on-a-chip, portal vein microfluidic systems, humanized mice, and paired primary-liver metastasis models can more authentically simulate the gut–liver axis and CRLM microenvironment. (5) Personalized microecological interventions should be promoted. Future intervention strategies should be stratified based on patients’ baseline microbiota, tumor molecular typing, immune status, and treatment plans, rather than employing a one-size-fits-all approach. Precision antibiotics, low-immunogenic bacteriophages, postbiotics, dietary interventions, and nanodelivery systems can be combined and applied according to different stages, but they must undergo rigorous safety evaluations.

Overall, research on the gut–liver axis microecology provides a novel theoretical framework for understanding the mechanisms and clinical translation of CRC liver metastasis. Microbial biomarkers are expected to aid in identifying individuals at high risk of liver metastasis, while circulating microbial DNA, bile acid profiles, and short-chain fatty acids may offer additional information for dynamic monitoring. Intervention strategies targeting the microecology, including precision antibiotics, phage therapy, postbiotic supplementation, and combined delivery technologies, also offer potential avenues for blocking metastasis and improving treatment responses. However, current evidence remains insufficient to support the direct integration of these strategies into routine clinical practice. Future research must further clarify the true contribution of the intestinal microbiome to CRLM, based on strict control of contamination, clear establishment of causal relationships, enhanced spatial localization, and prospective clinical validation. Only when microecological indicators can reliably improve risk prediction and microecological interventions can enhance patient outcomes under safe conditions can gut–liver axis strategies truly become integral components of precise prevention and treatment for CRC liver metastasis.

## 7. Conclusions

Colorectal cancer liver metastasis (CRLM) represents a complex organ-specific metastatic process involving continuous interactions among gut microbiota, intestinal barrier function, hepatic microenvironment, metabolism, and tumor cells. This review highlights the emerging concept that gut microbiota alterations are not merely associated with colorectal cancer progression but may actively participate in metastatic evolution through gut–liver axis-mediated regulation. Gut microbiota dysbiosis and barrier dysfunction facilitate the delivery of microbial components and metabolites to the liver through portal circulation, contributing to hepatic microenvironmental remodeling and the establishment of a pre-metastatic niche that supports subsequent tumor colonization. Beyond the pre-metastatic stage, microbial factors may continue to influence CRLM progression by regulating tumor cell adaptation, immune escape, metabolic reprogramming, and metastatic microenvironment maintenance. This perspective extends the traditional view of metastasis from a tumor cell-centered process toward a broader ecological interaction involving host tissues, microorganisms, and tumor cells. However, despite increasing evidence supporting the involvement of the gut–liver axis in CRLM, several important challenges remain, including insufficient causal validation in humans, limited longitudinal clinical evidence, technical difficulties in low-biomass microbiome analysis, and uncertainty regarding the safety and efficacy of microbiome-based interventions. Future studies integrating prospective clinical cohorts, spatial multiomics, metabolomics, and advanced computational approaches are required to clarify the temporal and functional relationship between microbiota alterations and metastatic progression. Well-designed clinical studies are also needed to determine whether microbiome-targeted strategies can improve early risk prediction, prevent metastatic development, or enhance therapeutic responses. Ultimately, a deeper understanding of gut–liver axis-mediated regulation may provide new opportunities for precision prevention and individualized treatment of CRLM.

## Figures and Tables

**Table 1:** Cellular Composition and Functional Characteristics of Hepatic Pre-Metastatic Niche (PMN).

Cell Type	Source/ Location	Key Activating Signals	Core Functions	Molecular Markers/Secreted Factors	Reference
Kupffer cells (liver-resident macrophages)	Hepatic sinusoids, liver	LPS/TLR4, MyD88, NF-κB	Recognizes gut-derived LPS and initiates sterile inflammation; Secretes pro-inflammatory cytokines and recruits myeloid cells; Functional impairment → reduced phagocytosis and collapsed immune surveillance	Secreted: TNF-α, IL-6, IL-1β, CCL2	[15,18,19]
				Phenotype: CD68+, TLR4+	[15,18,19]
Liver sinusoidal endothelial cells (LSECs)	Hepatic sinusoidal wall	LPS, DCA, inflammatory cytokines	Upregulates CXCL16 and regulates NKT cells; Increased permeability facilitates CTC retention; Expresses adhesion molecules (ICAM-1)	Secreted: CXCL16, ICAM-1, VCAM-1	[17]
				Receptors: CXCR6, TLR4	[17]
Hepatic stellate cells (HSCs)	Perisinusoidal space (Disse space)	TNF-α, IL-6, TGF-β, autophagy	Transdifferentiates from quiescent to myofibroblasts; Synthesizes extracellular matrix (ECM: collagen I, fibronectin); Matrix stiffening, CTC trapping and fibrosis promotion	Secreted: Collagen I, fibronectin, TGF-β, BMP-1	[20,21,53,54]
				Markers: α-SMA+, Desmin+	[21,54]
Neutrophils	Bone marrow → hepatic sinusoids	LPS, CCL2, CXCL5	Releases neutrophil extracellular traps (NETs) to physically trap CTCs; NETs shield NK/CD8+ T cells and promote immune escape; Pro-inflammatory and amplifies fibrosis	Released: NETs (DNA/histones/proteases), LDHA	[22]
				Markers: CD11b+, Ly6G+	[22]
Myeloid-derived suppressor cells (MDSCs)	Bone marrow → liver	CCL2, S1PR1–STAT3	Inhibits CD8+ T/NK cells; Induces NETs and synergistically promotes metastasis; Produces Arg1/iNOS, depletes arginine and generates NO	Secreted: Arg1, iNOS, TGF-β, IL-10	[12,22]
				Subsets: PMN-MDSC (CD11b+ Ly6G+Ly6Clow), Mo-MDSC (CD11b+Ly6C+Ly6G−)	[12,22]
Regulatory T cells (Tregs)	Thymus/periphery → liver	TGF-β, IL-10, lactate	Inhibits CD8+ T/NK cells; Maintains immune tolerance and protects CTCs	Markers: FOXP3+, CD25+, CTLA-4+	[12,22]
Natural killer (NK) cells	Liver, circulation	Fap2–TIGIT, lactate, MDSCs	Impaired CTC-killing capacity; Downregulated activating receptors (NKG2D/NKp30) and functional suppression	Receptors: T cell immunoreceptor with Ig and ITIM domains (TIGIT) ↑, NKG2D ↓	[23,24]
				Secreted: Perforin ↓	[23,24]

Notes: PMN, hepatic pre-metastatic niche; KCs, Kupffer cells; HSCs, hepatic stellate cells; MDSCs, myeloid-derived suppressor cells; NETs, neutrophil extracellular traps; Tregs, regulatory T cells; NK cells, natural killer cells. All listed molecules represent representative secreted factors or phenotypic markers of corresponding cells. ↑ indicates upregulation or increased expression/secretion; ↓ indicates downregulation or decreased expression/secretion.

## Data Availability

No new data were created or analyzed in this study. Data sharing is not applicable to this article.

## Abbreviations

The following abbreviations are used in this manuscript:

ABCB1	ATP-binding cassette subfamily B member 1
ABCG2	ATP-binding cassette subfamily G member 2
ALPPS	Associating Liver Partition and Portal vein ligation for Staged hepatectomy
Arg-1	Arginase 1
BFT	*Bacteroides fragilis* toxin
BRCA1	Breast cancer gene 1
BSH	Bile salt hydrolase
CCL	C-C Motif Chemokine Ligand
CCL2	C-C motif chemokine ligand 2
CDA	Cytidine deaminase
CEA	Carcinoembryonic antigen
CPBAs	Conjugated primary bile acids
CRC	Colorectal cancer
CRLM	Colorectal cancer liver metastasis
CTC	Circulating tumor cell
CXCL16	C-X-C motif chemokine ligand 16
CXCR6	C-X-C motif chemokine receptor 6
DCA	Deoxycholic acid
ECM	Extracellular matrix
ETBF	Enterotoxigenic *Bacteroides fragilis*
Fap2	Fusobacterium adhesin A
FAK	Focal adhesion kinase
FISH	Fluorescence in situ hybridization
*F. nucleatum* (Fn)	*Fusobacterium nucleatum*
FXR	Farnesoid X receptor
Gal-GalNAc	Galactose-N-acetylgalactosamine
G-MDSC	Granulocytic myeloid-derived suppressor cell
GVB	Gut vascular barrier
HSC	Hepatic stellate cell
iNOS	Inducible nitric oxide synthase
IFN-γ	Interferon-gamma
IL	Interleukin
KCs	Kupffer cells
LDHA	Lactate dehydrogenase A
LAG-3	Lymphocyte activation gene 3
LPS	Lipopolysaccharide
LSEC	Liver sinusoidal endothelial cell
MAPK	Mitogen-activated protein kinase
MDSC	Myeloid-derived suppressor cell
MDR1	Multidrug resistance protein 1
MGMT	O-6-methylguanine-DNA methyltransferase
MLCK	Myosin light chain kinase
Mo-MDSC	Monocytic myeloid-derived suppressor cell
MUC2	Mucin 2
MyD88	Myeloid differentiation primary response 88
NET	Neutrophil extracellular trap
NK cell	Natural killer cell
NKT cell	Natural killer T cell
NKG2D	Natural killer group 2 member D
NF-κB	Nuclear factor kappa-B
PAMPs	Pathogen-associated molecular patterns
PD-1	Programmed cell death protein 1
PDGF	Platelet-derived growth factor
PI3K/AKT	Phosphatidylinositol 3-kinase/Protein kinase B
PMN	Hepatic pre-metastatic niche
PV-1	Plasmalemma vesicle-associated protein 1
PXR	Pregnane X receptor
RAD51	RAD51 recombinase
RCTs	Randomized controlled trials
SCFA	Short-chain fatty acid
SEER	Surveillance, Epidemiology, and End Results database
SPP1	Secreted phosphoprotein 1
STAT3	Signal transducer and activator of transcription 3
TCA	Taurocholic acid
TGF-β	Transforming growth factor-beta
TGR5	Takeda G-protein-coupled receptor 5
TIGIT	T cell immunoreceptor with Ig and ITIM domains
TIM-3	T-cell immunoglobulin and mucin domain-containing protein 3
TLR4	Toll-like receptor 4
TNF-α	Tumor necrosis factor-alpha
Treg	Regulatory T cell
VE-cadherin	Vascular endothelial cadherin
Wnt	Wingless-related integration site
α-SMA	Alpha-smooth muscle actin
ZO-1	Zonula occludens-1
